# Modelling the interaction between stem cells derived cardiomyocytes patches and host myocardium to aid non-arrhythmic engineered heart tissue design

**DOI:** 10.1371/journal.pcbi.1010030

**Published:** 2022-04-01

**Authors:** Damiano Fassina, Caroline M. Costa, Stefano Longobardi, Elias Karabelas, Gernot Plank, Sian E. Harding, Steven A. Niederer

**Affiliations:** 1 School of Biomedical Engineering and Imaging Sciences, King’s College London, London, United Kingdom; 2 National Heart and Lung Institute, Imperial College London, London, United Kingdom; 3 Institute of Mathematics & Scientific Computing, University of Graz, Graz, Austria; 4 Gottfried Schatz Research Center (for Cell Signaling, Metabolism and Aging), Division Biophysics, Medical University of Graz, Graz, Austria; University of California San Diego, UNITED STATES

## Abstract

Application of epicardial patches constructed from human-induced pluripotent stem cell- derived cardiomyocytes (hiPSC-CMs) has been proposed as a long-term therapy to treat scarred hearts post myocardial infarction (MI). Understanding electrical interaction between engineered heart tissue patches (EHT) and host myocardium represents a key step toward a successful patch engraftment. EHT retain different electrical properties with respect to the host heart tissue due to the hiPSC-CMs immature phenotype, which may lead to increased arrhythmia risk. We developed a modelling framework to examine the influence of patch design on electrical activation at the engraftment site. We performed an *in silico* investigation of different patch design approaches to restore pre-MI activation properties and evaluated the associated arrhythmic risk. We developed an *in silico* cardiac electrophysiology model of a transmural cross section of host myocardium. The model featured an infarct region, an epicardial patch spanning the infarct region and a bath region. The patch is modelled as a layer of hiPSC-CM, combined with a layer of conductive polymer (CP). Tissue and patch geometrical dimensions and conductivities were incorporated through 10 modifiable model parameters. We validated our model against 4 independent experimental studies and showed that it can qualitatively reproduce their findings. We performed a global sensitivity analysis (GSA) to isolate the most important parameters, showing that the stimulus propagation is mainly governed by the scar depth, radius and conductivity when the scar is not transmural, and by the EHT patch conductivity when the scar is transmural. We assessed the relevance of small animal studies to humans by comparing simulations of rat, rabbit and human myocardium. We found that stimulus propagation paths and GSA sensitivity indices are consistent across species. We explored which EHT design variables have the potential to restore physiological propagation. Simulations predict that increasing EHT conductivity from 0.28 to 1–1.1 S/m recovered physiological activation in rat, rabbit and human. Finally, we assessed arrhythmia risk related to increasing EHT conductivity and tested increasing the EHT Na^+^ channel density as an alternative strategy to match healthy activation. Our results revealed a greater arrhythmia risk linked to increased EHT conductivity compared to increased Na^+^ channel density. We demonstrated that our modeling framework could capture the interaction between host and EHT patches observed in *in vitro* experiments. We showed that large (patch and tissue dimensions) and small (cardiac myocyte electrophysiology) scale differences between small animals and humans do not alter EHT patch effect on infarcted tissue. Our model revealed that only when the scar is transmural do EHT properties impact activation times and isolated the EHT conductivity as the main parameter influencing propagation. We predicted that restoring physiological activation by tuning EHT conductivity is possible but may promote arrhythmic behavior. Finally, our model suggests that acting on hiPSC-CMs low action potential upstroke velocity and lack of I_K1_ may restore pre-MI activation while not promoting arrhythmia.

## Author summary

Engineering heart tissue patches applied over myocardial infarcts have been proposed as a novel treatment option to augment cardiac electrical activation and contraction. Optimizing patches experimentally is challenging due to the large number of design variables and the need to work in small animal models, when humans are the target species. To address these challenges, in this study, we use virtual (computer) representations of heart tissue to simulate the electrical signal propagation and explore the effect of different patch designs. We simulated patches application on rat, rabbit and human myocardium, predicting that the experimental results obtained on small mammal can be representative of effects on larger (human) hearts. We identified tissue conductivity as the parameter most influencing the patch’s effect. Our simulations also showed that by modifying engineered tissues’ ion channels’ conductivities, electrical propagation similar to the one in the heart without scar could be restored, while avoiding the risk of abnormal heart rhythms.

## Introduction

Myocardial infarction (MI) hospitalizes 1 person every 5 minutes in the UK [1]. Although 7 persons out of 10 survive [2], their heart can be permanently compromised. Due to the lack of blood supply, MI can cause parts of the myocardium to become non-contractile and non-conductive, scar-like tissue. The presence of scar can have a severe impact on the heart, exposing patients to risks ranging from arrhythmias to impaired cardiac function.

The irreversible damage to the cardiac tissue caused by MI and the limited ability of the adult heart to regenerate lost cardiomyocytes (CMs) make cardiac transplantation or left ventricular assist devices (LVADs) the only treatments able to restore cardiac function. However, heart transplants are in limited supply and require life-long immuno-suppression [3], while LVADs have approximately 80% complication rate [4]. Cardiac regenerative medicine has recently been proposed as a promising alternative [3,5,6]. It comprises a wide spectrum of novel treatments, whose target is to replace or augment the function of tissue lost to cardiac infarcts. In this study we focus on the epicardial application of patches of stem cell-derived engineered heart tissue [7].

Many challenges need to be faced in the development of this treatment. A better understanding of the electrical interaction of the engineered and host heart tissue is crucial. While the presence of new engineered heart tissue can represent an alternative pathway for the electrical propagation in presence of scar tissue, the immaturity of the human induced pluripotent stem cells derived cardiomyocytes (hiPSC-CM) can lead to different electrical propagation properties to the host that may contribute to an increased arrhythmia risk [8–10]. *In vitro* experiments involving patches have been conducted using many experimental setups with differences in pacing rates, engineered heart tissue dimensions and host species, making it hard to compare results and interpret results in the context of human physiology [11–13].

Computer simulations of the heart have advanced to a point where they are being used for interpreting and guiding clinical procedures [14]. Here we apply this approach to develop and validate a tissue-scale model to simulate *in silico* electrical propagation in scarred heart tissue in the presence of a patch of engineered heart tissue and conductive polymer (CP) engrafted at the epicardium. We use machine learning and global sensitivity analysis techniques adapted to cardiac modelling to identify engineered heart tissue patch design variables that are important for restoring physiological electrophysiology in the host myocardium. We then test if these important variables are species dependent. Finally, we demonstrate how engineered heart tissues could be modified to recover physiological activation while reducing arrhythmic risk.

## Methods

### 1. Schematic tissue model

**Fig 1:**
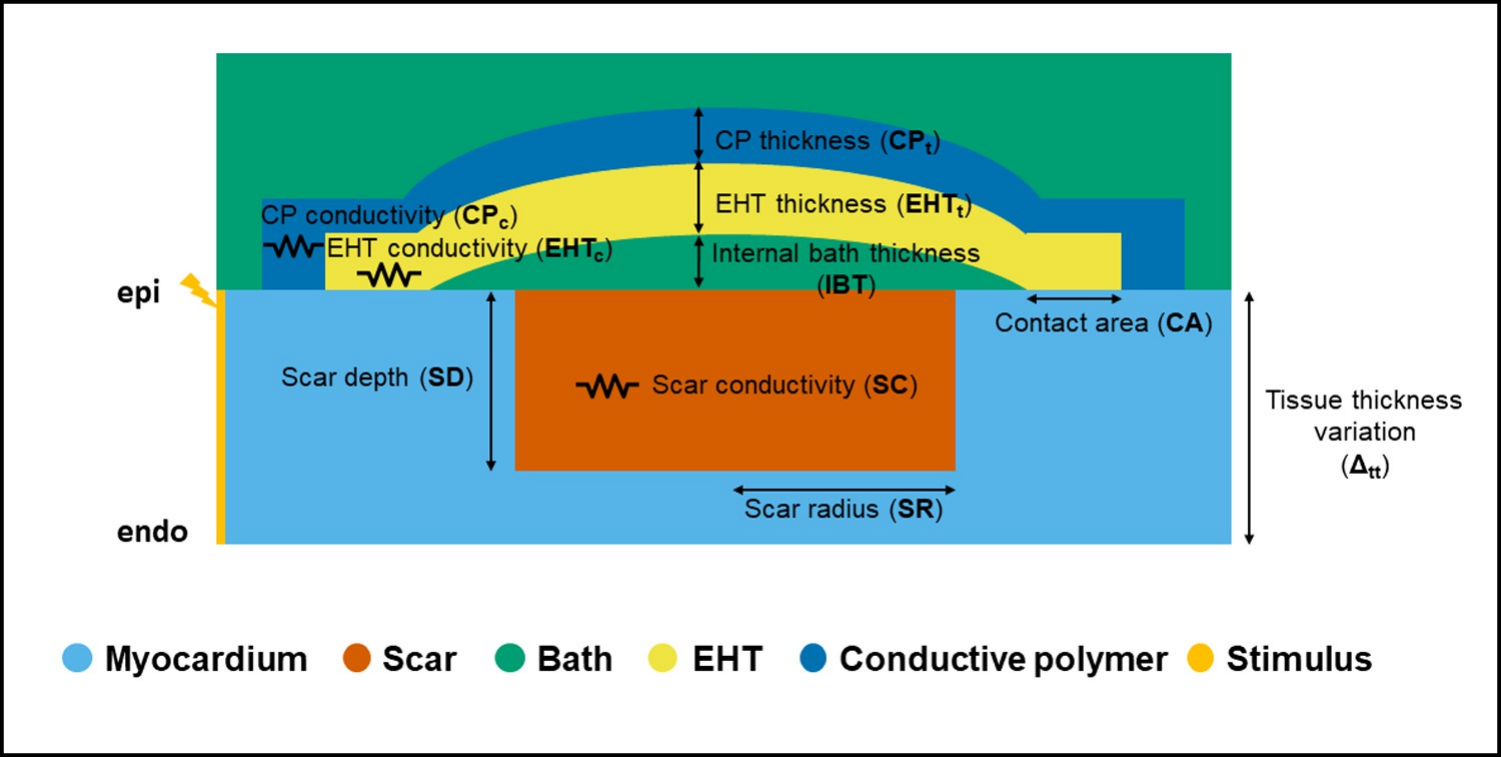
Schematic representation of our thin 3D tissue slab model along with the 10 design input parameters considered: Scar radius (SR), Scar depth (SD), Scar conductivity (SC), Engineered heart tissue thickness (EHT_t_), Engineered heart tissue conductivity (EHT_c_), Contact area (CA), Conductive polymer thickness (CP_t_), Conductive polymer conductivity (CP_c_), Internal bath thickness (IBT), Tissue thickness variation (Δ_tt_).

To investigate the impact of patch design on activation properties we created a thin 3D slab model of the myocardium (Fig 1), which approximates a scar in the left ventricle wall (Fig A in S1 Text). This model consists of a transmural cross section of host myocardium, an infarct region, a bath region, an epicardial patch spanning the infarct region and a space between the patch (designated the internal bath area) and host myocardium capturing imperfect contact due to attaching the patch to the host only at the edges. The patch is modelled as a layer of hiPSC-CM, which we refer to as engineered heart tissue (EHT) for brevity, combined with a layer of CP.

We consider 10 model input design parameters: tissue thickness (Δ_tt_), internal bath thickness (IBT), EHT thickness (EHT_t_) and conductivity (EHT_c_), CP thickness (CP_t_) and conductivity (CP_c_), scar radius (SR), transmural depth (SD) and conductivity (SC), and width of the EHT-slab tissue contact area (CA). Physiologically plausible ranges for each parameter were defined from the literature (Table 1). The tissue thickness was chosen based on literature values for the rat, rabbit and human hearts ventricular wall [15,16] (2, 4, and 6 mm, respectively), and variations from the reference value are encoded by the parameter Δ_tt_, ranging from -10 to +10% relative to the reference wall thickness value. To approximate the impact, if any, of incomplete engraftment, we set the internal bath thickness between 0.1 and 1 mm, although this range is not supported by literature values.

**Table 1:** Parameter ranges from literature for the 10 model input parameters.

parameter label	parameter definition	range	units	reference
SR	scar radius	0.7–6.6	mm	[21,22]
SD	scar depth	0.2–6	mm	[17,21,22]
SC	scar conductivity	0–0.14	S m^-1^	[23–26]
EHT_t_	engineered heart tissue thickness	0.5–2	mm	[5,8,17–20]
EHT_c_	engineered heart tissue conductivity	0.028–0.0224	S m^-1^	[5,8,17–20]
CA	engineered heart tissue-myocardium slab contact area	0.5–2	mm	[18–20]
CP_t_	conductive polymer thickness	0.5–2	mm	[27–29]
CP_c_	conductive polymer conductivity	0–18	S m^-1^	[27,28]
IBT	internal bath thickness	0.1–1	mm	-
Δ_tt_	variation in slab thickness	0–10	% of tissue depth	[15,16]

The values of the parameters related to the EHT patch (thickness, conductivity, and contact area) were chosen based on literature values from in vitro experiments where EHT patches had been engrafted onto the epicardium of infarcted hearts [5,8,17–20]. The EHT thickness range is 0.5–2 mm and the range for EHT conductivity was taken as 10–80% of healthy myocardial conductivity. The scar depth was chosen between 10% and 100% of the tissue thickness [17,21,22] (100% thickness corresponds to a transmural scar). The scar radius was set to between 0.7 and 6.6 mm. This allowed us to set the patch length to the average size of experimentally engrafted patches [21,22], while still covering the scar. The scar conductivity was varied between 0 and 50% of the healthy host myocardium conductivity [23–26]. The conductive polymer thickness was set to 0.5–2 mm and the conductivity values <18 S/m. Both values were gathered from in vitro measurements performed on conductive polymer patches [27–29].

**Fig 2:**
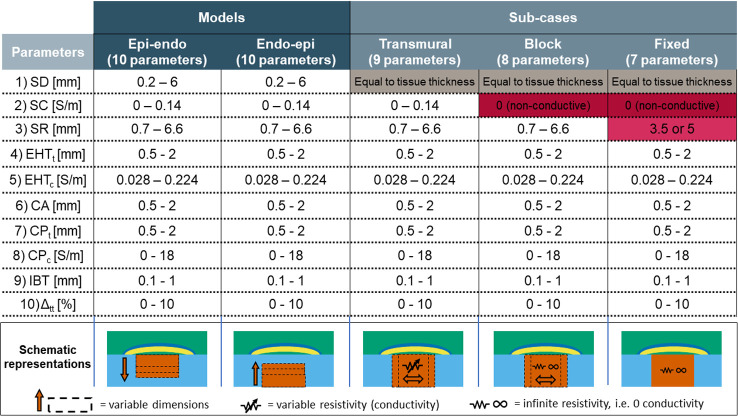
Value ranges of the 10 parameters (rows) used in the two versions of our model (Epi-endo, and Endo-epi, 1^st^ and 2^nd^ columns respectively). The 3 sub-cases (Transmural, Block and Fixed) are shown in the 3^rd^, 4^th^ and 5^th^ columns. The table shows which parameters of the original model are fixed in order to derive each sub-case. The bottom row displays representations of each model version and subcases.

We developed two versions of the model: one with the scar growing from the epicardium to the endocardium (labelled *epi-endo*), and one with the scar expanding from the endocardium to the epicardium (*endo-epi*). These two versions are described by the same 10 parameters. From these general models, we derived 3 sub-cases, progressively fixing the values of the 3 scar parameters (namely the scar depth, scar conductivity and scar radius). First, we set the scar depth equal to the tissue thickness, so that the scar is always transmural. This model was labelled *transmural*. Second, in addition to the scar depth being set equal to the tissue thickness, we also set the scar conductivity to 0, and we labelled this model *block*. Third, besides fixing the scar depth and conductivity, we also fixed the scar radius, setting it to 3.5 mm when modelling rat and rabbit myocardium and 5 mm when modelling human myocardium. We labelled this last sub-case *fixed*. Fig 2 below shows how the 3 sub-cases are derived from the two main versions of the model, as well as a schematic representation for each of them.

### 2. Simulating cardiac electrophysiology

Cardiac electrophysiology is simulated using the bidomain model [30]. The bidomain equations model the cardiac tissue as a syncytium, composed by the intracellular and the extracellular domains. Intracellular (*ϕ_i_*) and extracellular (*ϕ_e_*) potential are linked through the transmembrane current *I_m_*:

$$\nabla \cdot G_{i}\nabla \phi_{i}=\beta I_{m}$$

$$\nabla \cdot G_{e}\nabla \phi_{e}=-\beta I_{m}$$

Where *G_i_* and *G_e_* are the intracellular’s and extracellular’s conductivity tensors and *β* is the cardiac cells surface to volume ratio. The transmembrane current *I_m_* is expressed as follows:

$$I_{m}=C_{m}\frac{\partial V_{m}}{\partial t}+I_{ion}(V_{m},\nu )-I_{trans}$$

Where *C_m_* is the membrane capacitance per unit area, *V_m_* is the transmembrane voltage (defined as *ϕ_i_*−*ϕ_e_*), *I_trans_* is the transmembrane current density stimulus and *I_ion_* is the current density flowing through the membrane ion channels, which depends on the transmembrane voltage *V_m_* and other variables, summarized here with *ν*. The ionic cell model was chosen to represent rabbit [31], rat [32] and human [33] myocardium. For the EHT the model of hiPSC-CM electrophysiology from Paci et al [34] was utilized. The model equations were solved using the Cardiac Arrhythmia Research Package (CARP) [35].

In the cardiac tissue, myocytes are arranged in fibre-like structures, with propagation faster parallel to the fiber orientation than perpendicular to it [36]. To capture this anisotropic behavior, we incorporated fibers in our model, transmurally rotating (from endocardium to epicardium) from a 40° angle to a -50° angle, with respect to the longitudinal direction [37] (Fig B in S1 Text). The conductivities along the fibers’ direction (longitudinal) and perpendicular to the fibers’ direction (transversal) are set according to Roberts and Scher [36], for both the intracellular and extracellular domains (Table 2), leading to conduction velocities of approximately 50 cm/s in the longitudinal direction and 20 cm/s in the transverse direction. For all simulations the bath conductivity was set to 1 S/m, to match Tyrode’s solution conductivity [38,39]. We phenomenologically model the scar by decreasing the tissue conductivity [25]. No border zone between scar and healthy tissue or scar fibers remodeling were included in the model. The scar, EHT and CP conductivities are set for each simulation (ranges in Table 1). The scar conductivity range we set (0 to 50% of the healthy tissue conductivity) yields scar velocities between ~10 and ~40 cm/s, depending on the ionic model (rat, rabbit, or human). These settings allow our model to match the velocity range reported in the literature for propagation in scars, usually reported to be 50% or less than the healthy myocardial conduction velocity [40–43].

**Table 2:** Model quantities kept fixed in all simulations: conductivity of the internal and external bath areas, ionic model used for the EHT, conductivities and fiber orientations for the host myocardium.

definition	value	units	reference
EHT ionic model	Paci	-	[34]
longitudinal tissue conductivity	0.28	S m^-1^	[36]
transversal tissue conductivity	0.026	S m^-1^	[36]
fibers orientation	rule-based	-	[37]
longitudinal extracellular conductivity	0–22	S m^-1^	[36]
transversal extracellular conductivity	0.13	S m^-1^	[36]
bath conductivity	1	S m^-1^	[38,39]

All models are created using thin 3D tetrahedral slab meshes with an edge length of 100 μm. The reference cross section dimensions of the tissue simulations are 14x2, 14x4 and 20x6 mm for rat, rabbit and human, respectively, and are representative of the ventricular size and wall thickness in each species. Changes in wall thickness are encoded by a parameter (Δ_*tt*_), representing the change in wall thickness from the reference value. For analysis of the effects of geometry, we consider variations in scar length and depth, internal bath thickness, CP and EHT thickness and EHT-tissue contact (Fig 1 and Table 1).

The tissue is stimulated by injecting a transmembrane current (100 μA/cm^2^) on the left side of the slab, across the whole slab section (Fig 1). We simulate 350 ms of propagation to ensure the whole tissue is activated. For all species, the tissue was modelled as having a 1 Hz pacing rate to match standard experimental conditions. To approximate a steady state, the cell model was paced with a 30 μA/cm^2^ transmembrane current for 800 s at 1 Hz. The state variables were saved and were used for initializing the cell model in the tissue simulation.

### 3. Conductive polymer modelling

In previous works, charge transport in CPs has been modelled using a continuum assumption and partial differential equations (PDEs) to describe the rate of change in concentration of charged particles [44,45]. These studies aimed at the describing the ions and holes movement in a small portion (nanometers) of CP.

In this study we incorporate the CP in a myocardial slab model, modelling the CP on a larger scale (millimeters). To account for the effects of the conductive surface provided by the CP when in contact with the myocardium [28], we modelled the CP as an Ohmic conductor. This assumes that the charge concentration remains homogenous across the CP, that the concentration of charge carriers remains constant over the duration of the simulation, that the diffusive properties of the CP are homogenous, and that charge transport is governed by drift. The CP model is discussed further in the limitations section. The conductive properties are varied by the range of values reported in the literature (Table 1).

### 4. Stem cells-derived engineered heart tissue modelling

The structure of the patch is shown in Fig 1. The patch consists of two layers: a CP (blue) and a layer of hiPSC-CM (yellow) labeled as EHT. While the CP is modelled as an ohmic conductor, the EHT is modelled using the bidomain equations for cardiac tissue. The EHT connects the healthy myocardium at the two ends of the scar, effectively creating a new pathway for electrical propagation. The EHT is described by a hiPSC-CM ionic model to include features of non-mature cardiomyocyte phenotype, typical of hiPSC-CMs [46].

### 5. Gaussian processes-based emulators

The presence of MI scars creates regions of slow conduction in the heart, which can lead to arrhythmia [47]. To measure conduction delay caused by the patch, we computed the change in right epicardial activation time (REAT) in the model (measured as the time from stimulation to activation of the top right corner) and used this value to evaluate the impact of different parameters in our model.

Fully exploring the 10-dimensional design space is computationally intractable with bidomain simulations. Thus, to reduce the computational time to evaluate the model for a given set of parameters we created a Gaussian Process Emulator (GPE) for the REAT as a function of the model’s parameters (Table 1). In total, we trained 15 GPEs, one for each version of the model and one for each of the sub-cases, and for the 3 different species. Each emulator is composed by a deterministic part given by a linear regression model and a stochastic part given by a zero-mean Gaussian Process (GP). We used first-degree polynomials as basis functions of the linear regressor and considered the squared exponential and the Matern anisotropic kernels as the GP covariance functions [48]. We fitted the linear regressor coefficients first and then fitted the GP hyperparameters to the residual by maximal likelihood. We choose the coefficient of determination, or R^2^, to measure the accuracy of our emulator. The R^2^ score is computed with a 5-fold cross validation. The emulators were built using routines from *scikit-learn* [49], a Python open-source machine learning library.

### 6. Training set creation

To train the GPE we need a data set that maps from parameter values to the REAT value over the parameter space. For the 2 versions of the model (*epi-endo*, *endo-epi*), and each of the 3 sub-cases (*transmural*, *block*, *radius fixed*—see Section 2.1) we sampled 500 combinations of parameters from their n-dimensional parameter space using a Latin Hypercube (LH), with n = 10, 10, 9, 8, 7, respectively. At least 50 samples per parameter were considered. Simulations are performed for each parameter combination, leading to 7,500 simulations in total for training the GPE’s.

To post process the simulations, the activation times were computed from the transmembrane voltage, as the instant in which it crosses (with positive derivative) the –10 mV threshold.

The AP propagation across the tissue slab was classified into 3 different paths. Bottom: the wave travels in the lower half of the tissue slab. Middle: the wave travels in the upper half of the tissue slab. Top: the wave travels through the EHT.

### 7. Global sensitivity analysis

We performed a variance-based global sensitivity analysis (GSA)[50]. Specifically, we evaluate the total effect index, where the total effect for the i-th input parameter (STi) consists of the sum of the i-th first order effect (Si) and the higher-order interactions. The first order effect is a normalized index that explains how much output variance is explained by the i-th parameter on its own. The higher-order interactions represent the contribution to the output variance from the interactions of the i-th parameter with all the other input parameters [51].

### 8. Parameters that match healthy activation times

GSA highlights which parameters play a key role in stimulus propagation in our model. We selected the most important parameters to tune to recover physiological activation times in the model. The healthy activation times are estimated by removing the scar, the EHT and the CP patch from our model, reducing it down to a slab representing a transmural slice of host myocardium. We repeated this procedure for the rat, rabbit and human models. We compared the REAT for different parameters values in the models with an EHT patch with the corresponding simulated healthy activation times for each species.

### 9. Arrhythmic behavior

Finally, we focus on the human model and we assess whether the changes made to this model to match healthy electrophysiology led to pro-arrhythmic behavior. We chose to compute the repolarization gradients as a marker of arrhythmic activity. Early studies have established a close linking of repolarization heterogeneities and cardiac arrhythmogenesis [52,53]. Additionally, the presence of an imbalance between the current available and the current needed for cell excitation (source-sink mismatch) and repolarization heterogeneities has been linked with unidirectional block [54]. Since the engraftment of EHT patches is likely to introduce a source-sink mismatch (through the wavefront geometry and structural modifications), we considered the repolarization gradient to be, albeit approximately, a metric representative of the arrhythmic risk following EHT engraftment. To compute the repolarization gradients, we paced the model for 100 beats to reduce any transient effects. For the 100^th^ beat only, we calculated the time to 80% repolarization at each node of the mesh. We then calculated the magnitude of the gradient of these repolarization times. We report repolarization gradients in the tissue slab, focusing on the interface between the EHT patch and the host myocardium. We focused on this area as it was the area with the highest gradients, it was the area most affected by the presence of the EHT and was close to the border of the scar, which is an area that is prone to ectopics [55]. Spatial plots of repolarization times and gradients can be seen in Figs M and N in S1 Text.

### 10. Validation

To validate the model, we compared simulated changes in conduction velocity (CV) in the presence of non-native CMs engrafted on animal hearts in four optical mapping experiments [17,20,21,27]. All simulations use the model structured as described above (Fig 1), which we will refer to as reference model, with modifications to match the specific experimental setups of the four different studies (Table 3).

**Table 3:** Modifications to model parameters to match the experimental studies setups. The first row reports the ranges used in our simulation study, while the rows below display the parameters values used to replicate each of the 3 experiments.

	Model parameters									
Papers	SR [mm]	SD [mm]	SC [S/m]	EHT_t_ [mm]	EHT_c_ [S/m]	CA [mm]	CP_t_ [mm]	CP_c_ [S/m]	IBT [mm]	Tissue thickness [mm]
**Range used in this study**	0.7–6	0.2–6	0–0.14	0.5–2	0.028–0.224	0.5–2	0.5–2	0–18	0.1–1	1.8–6.6
**Jackman et al [21]**	-	-	-	1	0.224	0	-	-	0.1	3
**Thompson et al [17]**	-	-	-	0.2	0.224	30	-	-	0	0.2
**Zimmermann et al [20]**	5	3	0	3	0.224	2	-	-	0	3
**Mawad et al [27]**	3	2	0.06	-	-	-	0.5	16	0	2

In Jackman et al [21], Langendorff rat hearts in a bath were engrafted with a 10 mm x 10 mm patch of neonatal rat cells. Prior to engraftment patches were paced at 1 Hz. Whole hearts were paced at 2 Hz. CV was measured before and after engraftment, in 3 different locations: on the patch, on the tissue underneath the patch and on the tissue far away from the patch. To simulate their experimental setup, we removed the scar and CP from our reference model. We also set the EHT-tissue contact area to 0 to match the presence of a thin layer devoid of CMs between the patch and the epicardium, as highlighted by the histological exam. The remaining model parameters (second row in Table 3) were set based on their reference values (Table 1). We repeated the 1 Hz pacing protocol, first pacing the scar and then the tissue. We extracted activation times to compute CVs in the three locations where they were experimentally measured, i.e. patch, tissue under the patch and tissue far away from the patch. We use the Terkildsen-Niederer-Crampin-Hunter-Smith model [32] for rat electrophysiology and a neonatal rat cell ionic model [56] for the EHT.

In Thompson et al [17], rat neonatal cells monolayers are treated with growth factor β and superimposed with monolayers of human embryonic stem cell-derived cardiomyocytes. To replicate this setup, we removed scar and CP from the reference model, as done for the previous study, and set the tissue thickness to 0.2 mm, the internal bath thickness to 0 and the EHT-tissue contact area to 30 mm, i.e. the whole length of the monolayers (third row in Table 3). We set the bath conductivity to 0 S/m, since the monolayers were not perfused in a bath, but grown on fibronectin lines. We also reduced the tissue conductivity from 0.28 S/m to 0.123 S/m to match the 56% reduction in CV observed in the rat neonatal cells monolayers, following the treatment with growth factor β. The remaining parameters values were set as the mid values of the respective ranges, previously defined (Table 1). We used a neonatal rat cells ionic model [57] for the monolayers and the Paci model [46] for the stem cell-derived monolayers. We computed the rat neonatal cells monolayers CV before and after superimposing the stem cells-derived myocytes layer, as was done experimentally.

In Zimmermann et al [20], LAD ligation was performed on 8 Langendorff-perfused rat hearts, causing large transmural infarcts. The hearts were then engrafted with thick (15 mm x 15 mm x 2–3 mm) patches of neonatal rat cells. Myocardium CV was measured before and after engraftment through multi-electrode epicardial mapping. This system was modelled by removing the CP from the model and by setting the EHT thickness to 3 mm. Values belonging to the previously defined ranges were used for the remaining parameters (fourth row in Table 3). CV was again computed on the myocardial slab, before and after adding the EHT patch, and predicted changes in CV were compared against experimental observations.

In Mawad et al [27], polyaniline patches were engrafted on both healthy and infarcted Langendorff-perfused rat hearts. CVs were computed through optical mapping data on healthy and infarcted hearts, before and after patch implantation. The CP patches did not include cardiomyocytes, for this reason we removed the EHT from our reference model. We then set the CP patch conductivity to 16 S/m, to match the one reported in the paper. We also removed the scar to model the healthy hearts, and we paced our model without (control) and with the CP patch. Finally, we added back the scar to the model, tuning the scar conductivity to match the decrement in CV observed in the *ex-vivo* hearts after infarction (fourth row Table 3). The resulting CV was taken as control. We then added the CP patch to the model with the scar, paced and measured the CV.

The exact nodes used to compute the CVs, as well as schematic representations of all the models used can be found in Fig C in S1 Text.

## Results

### 1. Model validation

**Fig 3:**
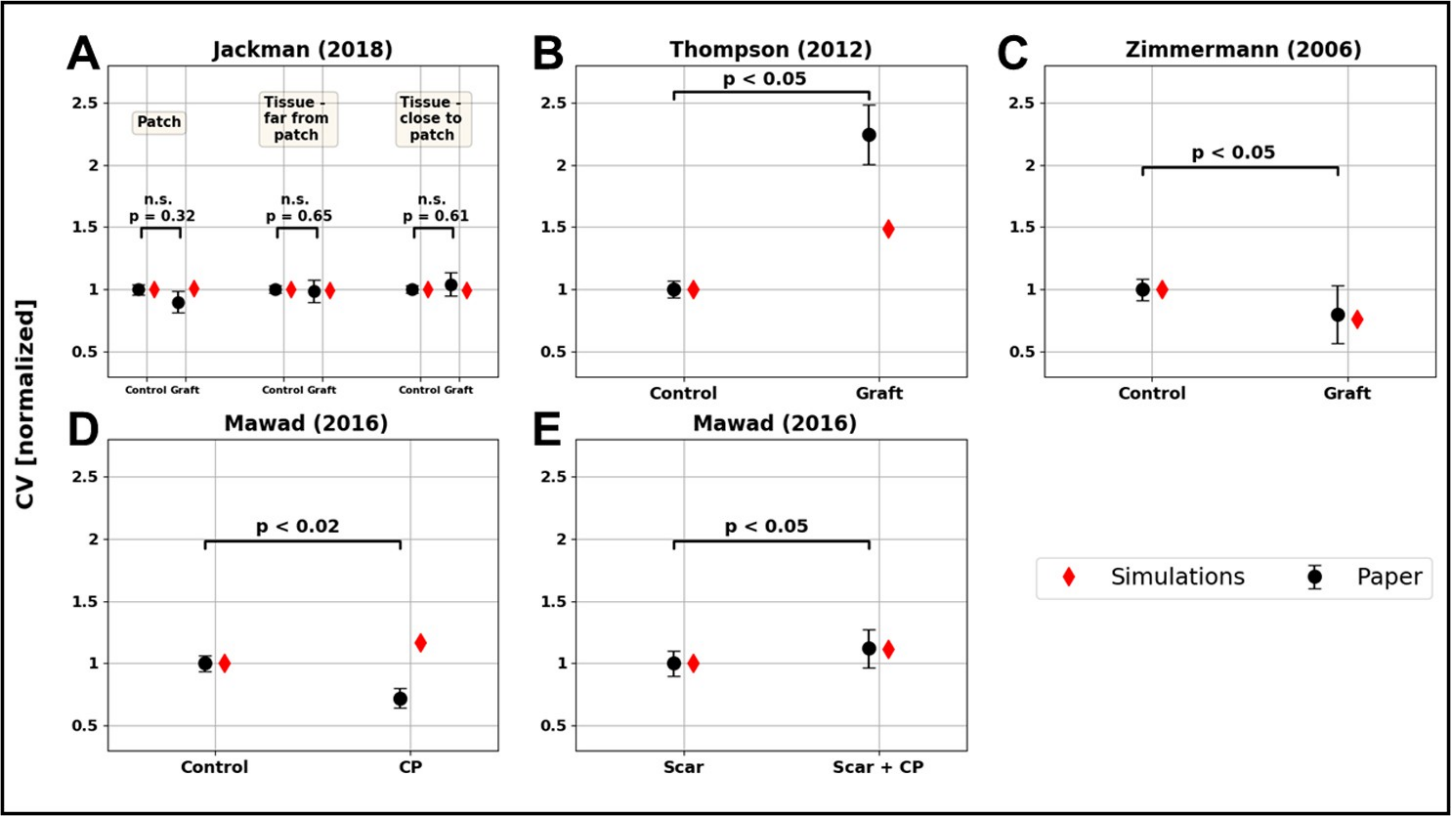
Results of the model validation. Experiments from 4 papers were compared to the model: Jackman et al (A), Thompson et al (B), Zimmermann et al (C) and Mawad et al (D-E). In panel A CV is evaluated in 3 different areas: on the patch, on the tissue far from the patch and on the tissue under the patch. In all 3 experiments, CV before (control) and after (graft) the attachment of the patch is compared. The CV measured experimentally (mean and standard deviation) are represented as black dots with error bars, with the p-values associated with each experiment. The simulated CV are displayed as red diamonds.

We compared simulations results against four previously reported experimental studies [17,20,21,27]. Consistent with Jackman’s finding of no significant change in the CV, our model predicts no change (Fig 3A). Using our modeling framework, we predict an increase in CV of 20%. This compares with a 110% increase in CV reported by Thompson et al, (Fig 3B). Our modelling framework captures this increase in CV qualitatively, if not quantitatively. This demonstrates that our modelling framework behaves as expected. However, a model calibrated for specific tissue patches is likely to be necessary to achieve quantitative agreement with experimental results. For this reason, in subsequent analysis, we account for the potential uncertainty in the model parameters by performing a global sensitivity analysis over ranges of values, as opposed to specific parameter values. Zimmermann et al, reported that the host CV after patch implantation was 20% lower than in healthy hearts, this compares with a 22% decrement in CV in our simulations (Fig 3C). Mawad et al, reported a decrement in host CV after CP patches grafting. This observation was not matched by the model, that predicted a ~10% increment of CV (Fig 3D). However, in a similar experiment in scarred tissue Mawad et al reported a 10% increment, that was matched by the simulations (Fig 3E).

### 2. Do the effects of engineered heart tissue on activation depend on the species?

Current experiments for evaluating EHT in infarcted hearts use small animal models, predominantly rabbit and rat [11]. To test if specific patch design variables change with different species, we compare the simulations in models with rat, rabbit and human host myocardium. We first consider the stimulus propagation paths. Secondly, we compute the GSA total effect indexes to highlight the most important parameters.

#### 2.1 Distribution of stimulus propagation paths

**Fig 4:**
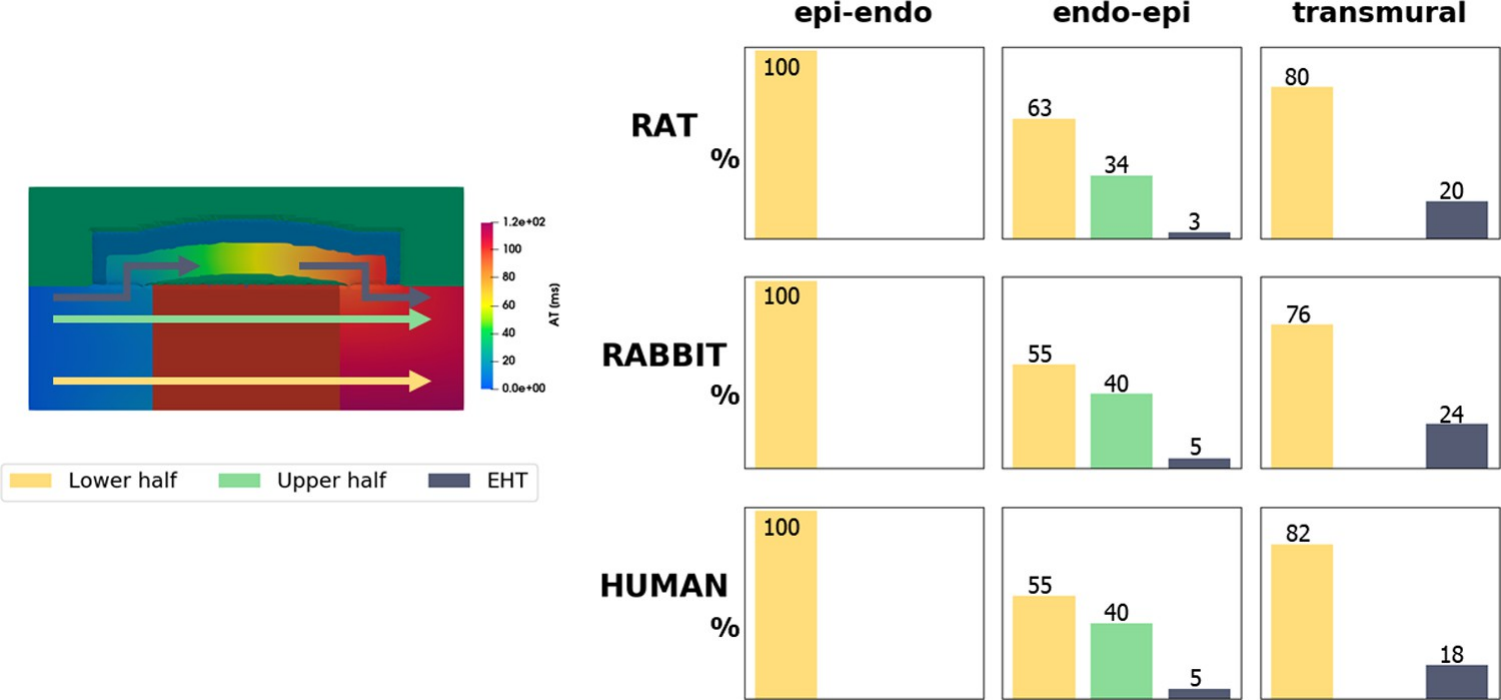
Cross-species comparison of the 500 simulations run to create the training datasets, classified as lower half, upper half or EHT, according to the propagation path (arrows in the left panel) followed by the stimulus. The colours on the right panel represent the arrows colours in the left one. Yellow, green and blue columns show the percentage of simulations classified as lower half, upper half or EHT, respectively. The percentage values are reported on each column.

We assessed if the path of activation dynamics in the presence of a scar and patch are consistent across the 3 species. We classified the activation wave path across the model in all simulations (500 for each of the 5 model setups–see sections 2.1 and 2.6). The activation wave path is classified based on whether the stimulus propagated through the lower half of the slab, the upper half of the slab, or through the EHT (Fig 4). In rat, rabbit and human, in the *epi-endo* setup, the stimulus always reaches the right side of the slab propagating through its lower half. In the *endo-epi* setup, a decrement of cases classified as “lower half” is observed across all the 3 species, together with propagations both through the upper half and the EHT. In the *transmural* setup, stimulus spreading is divided between lower half and EHT in rat, rabbit, and human models. *Block* and *fixed* setup are not shown, as the stimulus is forced to travel through the EHT. This shows that, in our study, the dimensions and electrophysiology of each species does not impact the activation wave propagation path.

#### 2.2 Impact of model parameters on stimulus propagation

**Fig 5:**
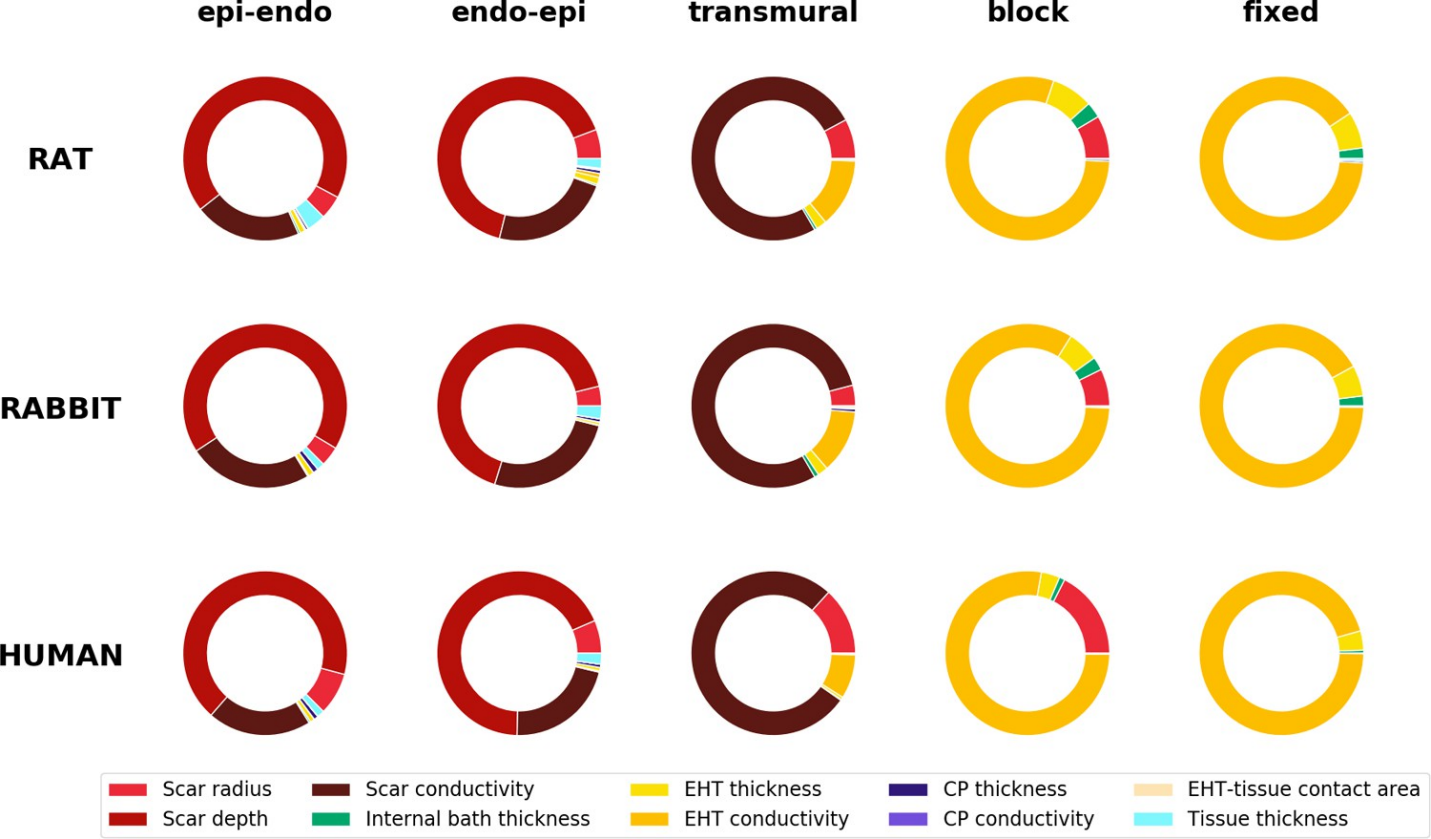
Donut charts representing the total effect index from the GSA. The figure compares the total effect indices obtained from the rat, rabbit, and human models (rows), for the epi-endo and endo-epi versions of the model and the transmural, block and fixed model sub-cases (see section 2.1).

In Fig 5 the total effect indexes from the GSA for each of the 5 setups (see section 2.1) for the rat, rabbit, and human model are plotted. For all species, the indices indicate that in the first 3 setups (*epi-endo*, *endo-epi* and *transmural*) the stimulus propagation is mainly affected by the scar parameters. Specifically, in the first 2 setups (*epi-endo* and *endo-epi*) the scar depth and the scar conductivity have the highest indexes, explaining between 66–70% and 21–27% of the model output variance in the first setup and 65–69% and 20–25% in the second one. In the third setup (*transmural*), where the scar depth is fixed, the variance is mostly explained by the scar conductivity (76%). The EHT conductivity also starts to play a role. In the last two setups (*block* and *fixed*), when the activation wave is forced to propagate through the EHT, the sensitivity indexes indicate that the EHT conductivity explains 80% and 90% of the output variance in the *block* and *fixed* case, respectively. Comparing all 5 setups, we can observe that for all species the total effect index identifies the scar parameters as the most important for output variance in the *epi-endo*, *endo-epi*, and *transmural* setups. EHT conductivity is also confirmed in all species as the parameter explaining most output variance in the *block* and *fixed* cases. Finally, neither of the two CP quantities considered (thickness and conductivity) showed a high sensitivity index, despite the wide range of CP conductivities explored (Table 1).

### 3. Parameter values that match healthy electrophysiology

**Fig 6:**
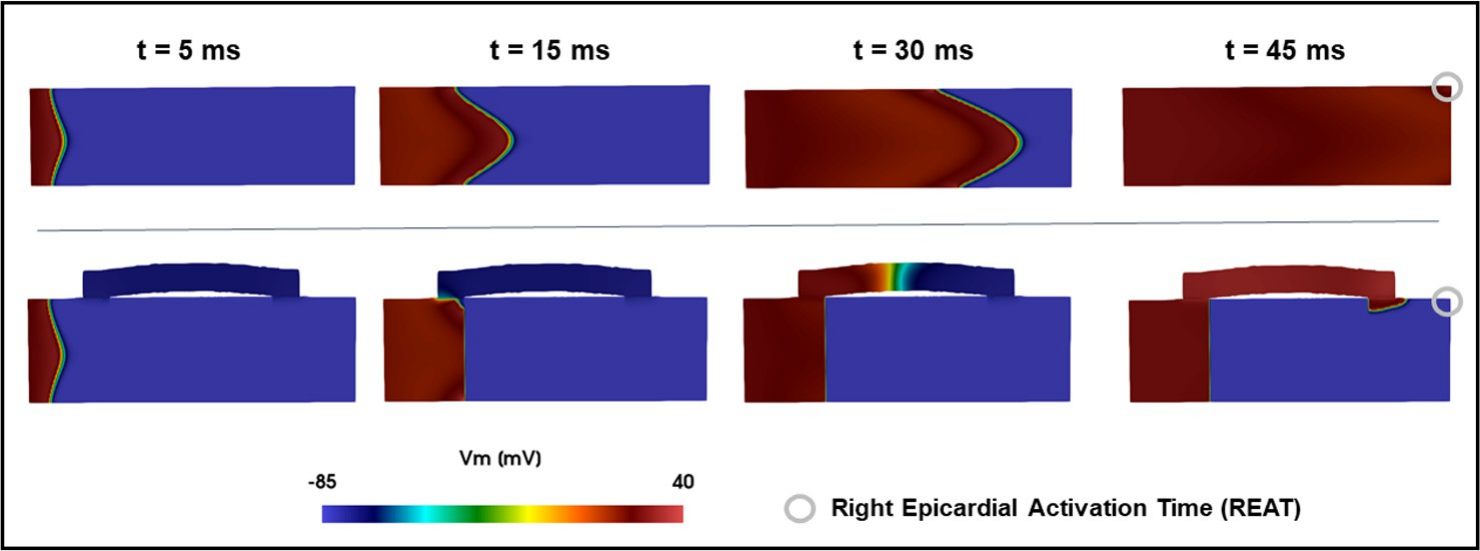
Sample activation pattern. Upper row: propagation in a healthy slab. Lower row: propagation in a sample simulation of the fixed setup, showing the delay in REAT activation.

In models of the rat, rabbit, and human we simulated electrical activation in healthy myocardial slabs in the absence of scar or EHT. For the rat, rabbit and human models, the time for the activation to spread across the model tissue to the top right corner was 35.3, 34.9 and 45.8 ms, respectively. This provided our baseline target healthy REAT values (Fig 6).

**Fig 7:**
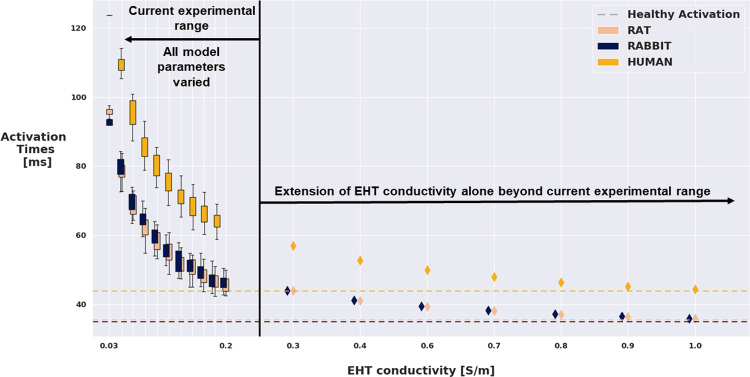
Model output (REAT) VS EHT conductivity for the rat, rabbit and human model, in the fixed setup. Boxplots represent the distribution of REATs among the 500 LH simulations that had the EHT conductivity restricted to current experimental ranges. Diamonds represent REATs obtained by fixing all the other parameters and increasing EHT conductivity by multiplying the upper bound of the range used in the LH by 1.5, 2, 2.5, 3, 3.5, 4 and 4.5 folds. Dashed line indicates the healthy REAT estimated by our models.

In all simulations performed with scar, the presence of EHT did not lead to physiological activation times, considering an interval of +/-10% of the healthy target REATs (Fig 6). In the *blocked* and *fixed* models the GSA (Fig 4) identified EHT conductivity as the parameter explaining most of the variance in simulated activation times in all the 3 species. Following these results, we tested whether increasing EHT conductivity beyond reported experimental bounds could bring the activation time back to physiological values in the *fixed* model (Fig 7). For all species, the EHT conductivity was increased 1.5–5 fold, with respect to the upper limit for the reported experimental range chosen in the LH sampling (Table 1). For all the 3 species, a minimum 3-fold increase in the EHT conductivity (equal to 0.672 S/m) would be needed to bring the activation time to within +/- 10% of the physiological value. The rat and human models achieved healthy activation times with EHT conductivity multiplied by a factor of 4.5, equal 1.008 S/m, while the rabbit model required the EHT conductivity to be multiplied by a factor of 5, equal to 1.12 S/m.

### 4. Achieving physiological activation time with low arrhythmia risk

Due to the hiPSC-CMs immature electrical properties, engraftment of EHT patches of hiPSC-CMs may induce arrhythmias [8,9]. Thus, we investigated whether the proposed patch designs exhibit pro-arrhythmic behavior in the human model. Focusing on the *fixed* setup, as this had the smallest number of degrees of freedom, we considered repolarization gradients as a surrogate for arrhythmia marker [26]. Specifically, we paced our tissue model 100 times, to limit transient effects, and computed and analyzed the repolarization gradients for the final beat. We focused on gradients in the host myocardium at the interface with the patch, where the contact between the human myocardium and the EHT is more likely to trigger pro-arrhythmic behavior.

We considered the model with EHT conductivity equal to 0.112 S/m (the mid value in the experimental range) as the baseline case, where the propagation is slower than the physiological one. The baseline case exhibits a mean repolarization gradient of ~18 ms/mm and no ectopic beats (Fig 7).

We then considered the case with EHT conductivity equal to 1.008 S/m, the value needed to match healthy activation time. In this case the mean repolarization gradients increased to ~50 ms/mm but did not cause ectopic beats.

Our initial analysis (Fig 5) did not consider ion channels in the sensitivity analysis. To increase CV without increasing repolarization gradients, we increased Na^+^ channel density in the ionic model used to represent hiPSC-CM in the EHT patch [46], while keeping the EHT tissue conductivity at the baseline value (0.112 S/m). This new model exhibited healthy activation times when the default Na^+^ channel density was increased by a factor of 4. The repolarization gradients were similar to the baseline value (~18 ms/mm). However, the increment in Na^+^ channel density caused a shortening of the hiPSC-CMs cycle length and resulted in ectopic beats within the EHT patch.

**Fig 8:**
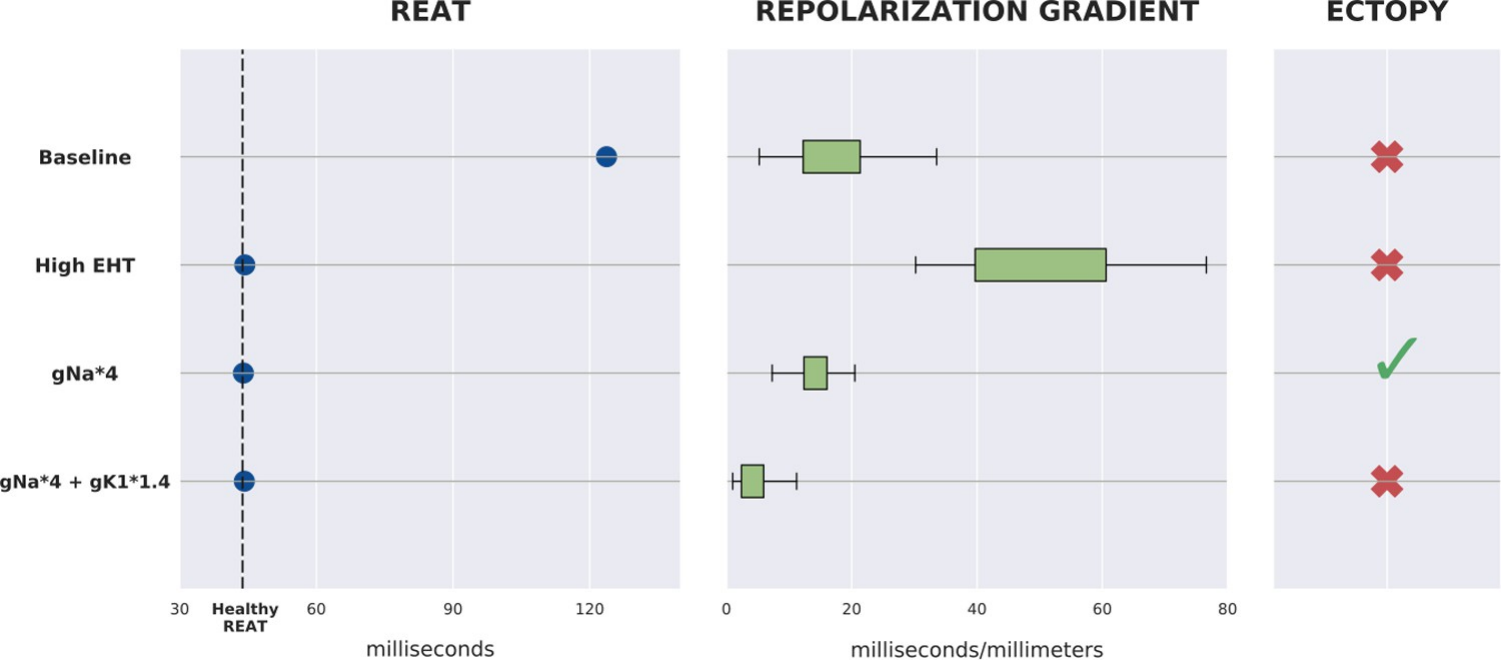
Summary of the 4 cases tested for arrhythmic behavior. For the Baseline and High EHT cases, EHT_c_ is set to 0.112 S/m (mid value in experimental range) and 1.008 S/m, respectively, and no modifications to ion channels’ densities are made. When the density of the ion channels responsible for the fast Sodium current and the inward rectifier potassium current are increased, the EHT_c_ is set to 0.112 S/m.The left panel shows which cases match estimated healthy REATs. The center panel shows the distribution of the mean repolarization gradients at the interface between myocardium and EHT (on 100 beats pacing protocol). The right panel shows the presence or absence of ectopic beats fired from the EHT.

The inward rectifier current (I_K1_) plays a key role in regulating hiPSC-CM self-pacing behavior [58,59]. We thus increased by a factor of 1.4 (from 28.2 nS / pF to 39.5 nS/pF) the density of the ion channels responsible for the potassium ions movement associated with the inward rectifier current. This resulted in hiPSC-CM cycle-length lengthening, which successfully ended the EHT-generated ectopic beats. The repolarization gradients also decreased, exhibiting a value of ~4 ms/mm, lower than the baseline case (Fig 8). For a systematic analysis of the effects of different gNa and gK1 values in the EHT refer to the dedicated section in S1 Text.

## Discussion

In this study we implemented an *in silico* cardiac electrophysiology model to investigate the impact of epicardial EHT patch design on the electrical propagation within infarcted myocardium. Tissue and patch geometrical dimensions and conductivities were incorporated through 10 modifiable model parameters (Fig 1). We demonstrated that our modeling framework could capture the interaction between host and EHT patches. We found no difference in the impact of EHT on activation or the importance of different patch design values between species models. We demonstrated that only when the scar is transmural do EHT properties impact activation times. Finally, we predicted that increased sodium (I_Na_) and potassium (I_K1_) channel densities in the EHT properties will lead to recovery of activation times, no change in repolarization gradients and no ectopy. This provides a testable prediction for recovering host electrophysiology properties using EHT patches.

### 1. Model validation

In our validation study, the modeling framework was able to qualitatively match the changes in CV observed in all but one experiment in Mawad et al (Fig 3D), where our simulations predicted an increment in CV after engraftment, instead of a decrement, as reported in the study. Since we are modelling the CP as an ohmic conductor (see section 2.3), this increment in CV is expected and comes as a result of the bath loading effect [60]. Essentially, the presence of a conductive area around the tissue increases the extracellular conductivity for the tissue at the interface, supplying the current with a low-resistance pathway to flow in. This is an experimentally documented phenomenon [61], which is reproduced by the bidomain model. The bath loading effect may be responsible for the increment in CV in Mawad et al when the same CP patch was implanted on infarcted hearts (Fig 3E). Mawad et al also recorded slowing in CV after engrafting CP patches on myocardial slices. Crucially, after the patch was removed, the myocardial slice did not recover to the CV observed before the engraftment, suggesting that epicardial or myocardial damage could potentially be involved in the observed CV slowing.

### 2. Conductive polymer effects

Attaching conductive polymer to EHT has been proposed as way to reduce arrhythmia risk [62]. The model captured the effects of the conductive polymer on infarcted hearts (Fig 3E). However, the GSA did not predict a role for the CP in impacting the REATs (Fig 5). While CPs proved useful as a culturing surface, allowing pacing and synchronization of multiple cluster cells by propagating electrical stimuli [29], these experimental studies and our simulation results do not provide clear support for their role in increasing CV in EHT patches.

### 3. From small animal models to human models

The species most commonly used in preclinical studies consist of small mammals such as rats and rabbits [63], which have distinct electrophysiology from humans [64]. Specifically, while some ionic currents such as I_Na_, I_K1_ and are conserved between species, other currents like the ones involved in the calcium handling (I_CaL_, I_NaCa_ and the Na-Ca exchanger) and the repolarizing currents (I_Kr_, I_Ks_) have found to be systematically different across species [64]. Current experiments for evaluating EHT in infarcted hearts are also mainly performed on small mammals. Testing whether findings in small animals models are transferrable to humans is a crucial step towards clinical application of EHT patches [11]. Consistent with previous simulation studies [65], we demonstrate that small animals provide a good model for replicating the qualitative effects of EHT patches on electrophysiology and that EHT design variables have similar importance and impact across species.

#### 3.1 Distribution of stimulus propagation paths

The majority of simulations paths are classified as “lower half” (Fig 4). This is likely caused by the fiber distribution. The fibers angle ranges from 40° to -50°, thus the longitudinal direction is more aligned with the conduction direction in the lower half of the slab. Our model also indicates that the distribution of stimulus paths is consistent across species. This result shows that the different electrophysiology of rat, rabbit and human CMs does not influence the electrical propagation in cardiac tissue in presence of a scar and an EHT patch. While some findings on small animal models, such as drugs effects, needs to take into account different between species at a cell level, such as ion channel expression [66], our results suggest that for electrical propagation the findings obtained on small animal models are transferrable to larger human models.

#### 3.2 Sensitivity indexes from GSA

Fig 5 indicates that the activation in the *epi-endo* and *endo-epi* setups is governed by the scar parameters (depth and conductivity), suggesting that the presence of EHT in our model does not have an impact when the scar is not transmural. In the *transmural* setup, however, the EHT conductivity starts to play a role. These findings are consistent with the stimulus path investigation (Fig 4), indicating the stimulus travelling almost exclusively through the slab in the *epi-endo* and *endo-epi* setups and travelling through the EHT in 18 to 24% of the simulations in the *transmural* setup.

The sensitivity indexes are consistent across rat, rabbit and human, indicating that the parameters which mostly influence propagation are not species dependent. These findings highlight the translational potential of the small animal studies, while encouraging, where possible, the use of computer model to back/substitute experimental studies on animals.

### 4. Restoring healthy activation while minimize arrhythmia risk

We found that conduction was slower in EHT than healthy tissue, this was further exacerbated by the need for the activation wave to move up into the EHT and down into the viable tissue. This increases wavefront curvature, which can further slow activation [67]. We predicted that EHT conductivity in the human model needs to be increased to 1.008 S/m (corresponding to 4.5 times the healthy myocardial conductivity) to match physiological activation patterns. Improving conductivity in cardiac tissue is an area of active research, and can be accomplished through techniques such as preconditioning with insulin-like growth factor-1[68], the delivery of the skeletal muscle Na^+^ channel through mesenchymal stem cells [69,70] and the gene transfer of connexin 32[71]. However, the lower conductivity of EHT is known to be caused by a combination of factors arising from the hiPSC-CMs immature phenotype [72,73]. Specifically, hiPSC-CMs present a lower expression of gap junction proteins with respect to adult cells. Moreover, gap junctions in hiPSC-CMs are arranged in a more circumferential distribution, like that in fetal cardiac cells, rather than being abundant at the longitudinal ends of the cell, as it happens for mature CMs [74]. Improving hiPSC-CMs maturation could address these issues, provide hiPSC-CMs with more adult-like gap junctions and thus bring EHT conductivity close to the values proposed here. Experimentally, increasing the expression of gap junction proteins and development of parallel sarcomeres have been achieved through extending the culture time [75] and electrical [76] or mechanical [77] stimulations. Although this is a long and difficult process, latest studies have shown encouraging developments in hiPSC-CMs maturation techniques, on a molecular and metabolic level [72], as well as regarding structural and functional maturation of hiPSC-CMs derived tissues [78]. New culturing techniques are also being developed to be easily scalable, and being to produce a large number of cells, required for tissue manufacturing and transplantation [73].

#### 4.1 Increasing EHT conductivity promotes pro-arrhythmic behavior

Our results indicate that increasing conductivity causes a rise in repolarization gradient at the myocardium-EHT interface (Fig 8). This can be explained by the increment in EHT electrotonic load on the tissue. The electrotonic load is due to diffusive currents flowing through the gap junctions of neighboring cells [79]. A large increment in conductivity, while restoring physiological activation times, could also exacerbate current diffusion between cells. Electrotonic currents are associated with changes in AP amplitude, AP repolarization and action potential duration (APD) [79,80] (for visualization of this phenomena in our model’s AP traces see Fig O in S1 Text). In turn, heterogeneities in AP repolarization are linked to the formation of arrhythmogenic substrates [54]. Hence, our findings suggest that achieving the required CV through (large) augmentation of EHT gap junctions density may promote arrhythmic behavior at the myocardium-patch interface.

#### 4.2 Altering ion channel densities recovers healthy activation and has low arrhythmic risk

Our model predicts that increased sodium (I_Na_) and potassium (I_K1_) channel densities in the EHT match the physiological activation patterns with no ectopy (Fig 8). Experimentally it has been shown that the immature phenotype causes hiPSC-CMs to have a lower AP upstroke velocity with respect to adult CMs [81]. This further hampers the possibility for hiPSC-CMs to reach CV values typical of adult CMs. The AP upstroke velocity is known to be mainly regulated by the Na channel density [67]. Our model estimates a 4-fold increase in I_Na_ conductance in the Paci model (from 3.67 nS / pF [34] to 14.68 nS/pF) to be sufficient to match physiological activation. The augmented value is more in line with Na channels density values found in adult CMs [67]. This finding further highlights the importance of hiPSC-CMs maturity when it comes to successful electrical integration with host tissue. The low I_Na_ expression also provides an additional explanation for the high values of EHT conductivity needed, according to our model (Fig 7), to match healthy activation. If the low Na^+^ channel density is retained, the EHT conductivity will have to be largely increased through gap junction density augmentation, which our model predicts to be pro-arrhythmic.

We observed in our model that the increment in I_Na_ caused a shortening of the hiPSC-CMs cycle length, resulting in the EHT firing ectopic beats (Fig 8). This is consistent with a computational study from Paci et al where they found a decrement in cycle length when substituting the adult formulation of I_Na_ from the O’Hara model [82], which has higher I_Na_ conductance, in the first version of their hiPSC-CMs model [83].

In addition, our model exhibited ectopy termination when, beside I_Na_, also I_K1_ was increased 1.4-fold, leading to physiological activation recovery, no increase in repolarization gradients and no ectopy. This is in agreement with earlier experimental works, which reported that introduction of adult-like I_K1_ eliminated hiPSC-CMs spontaneous activity [84] and brought hiPSC-CMs response to certain drugs closer to the one of adult CMs [85]. Low levels of the KCNJ2 gene in hiPSC-CMs is responsible for low I_K1_ expression and it is known to be one of the features that makes the hiPSC-CMs phenotype immature [86]. Thus, our findings place further emphasis on the importance of hiPSC-CMs maturation in the success of EHT patch engraftment.

## Limitations

In this study we used an idealized thin 3D tissue model that does not include realistic whole heart structure or geometry. Our model cannot therefore capture possible phenomena arising from the 3D structure and complexity typical of the mammal ventricles. To test if tridimensionality could affect our results, we have run further tests (reported in S1 Text) and verified that the GSA sensitivity indices do not change when considering the possibility of activation around the scar, and that the repolarization gradients do not change in a full-3D slab model.

A lower bound of 0.1 mm for the internal bath thickness parameter was set to maintain a consistent mesh resolution among the automatically generated models. In S1 Text, we show how a gap of 0.1 mm is nearly equivalent to the 0 mm case, introducing a maximum of 0.7% variation in the activation time.

When creating our model, we made a number of assumptions and parameters choice, as happens for every modelling study. To test if these choices impacted our results, we have used the rat model, *fixed* sub-case, as a reference model and repeated our GSA for different conditions. Specifically, we have tested: 1- Pacing location; 2- Pacing frequency; 3- Presence of border zone (BZ); 4- Transmural heterogeneity of ventricle adult cardiomyocytes. Results of these additional tests (reported in S1 Text) show a maximum variation of 3% in the computed sensitivity indices, suggesting that the assumptions made do not affect our findings.

We used a simplified Ohmic conductor model of the CP. In more detailed modelling studies, Wang et al [45] matched experimental measures on CP by modelling drift and diffusion of charged species with Nernst-Plank equations. However, their results did not change when neglecting charges diffusion, concluding that the influence of diffusion in charge transport in CPs is debatable. In another study, Cochrane at al [87] have measured the current/voltage (I/V) relationship in CPs, and demonstrated that it becomes more linear (i.e. it follows the Ohm’s law) as the conductive particle concentration (doping) increases. Therefore, the modelling strategy adopted in this study (CP as an Ohmic conductor) empirically captures CP’s bulk behavior, but it is not representative of the CP’s charges mechanism at a molecular scale.

We modeled the EHT as a homogenous syncytium. However, it is likely that EHTs contain stem-cell-derived cardiomyocytes exhibiting three phenotypes: atrial-like, ventricular-like, and nodal-like [88,89]. We have not represented these distinct cell types explicitly and have assumed a single homogenized representation for the EHT. The need to explicitly represent different cell types depends on their relative number and their spatial organization. This is likely to depend on the EHT manufacturing process and could be modeled using the partitioned phenotypes or homogenized phenotype models for simulating electrical activation in tissue consisting of multiple cell types [90].

We used repolarization gradients as an arrhythmia marker, however, Laurita et al [54] found that heterogeneities in AP repolarization are linked to the formation of arrhythmogenic substrates when an isthmus is present, which we did not explicitly include in our model. However, when the activation wave moves from the EHT and down into the viable tissue it will lead to enhanced wave curvature, which Laurita et al also listed as an alternate to an isthmus as a possible source of electrotonic load. Other studies have also shown that increased dispersion of repolarization enhances the occurrence of unidirectional blocks [26], highlighting that, alongside structural, also functional reentry may be possible.

Finally, in this study we have modelled the scar as non-conductive tissue or tissue with lower conductivity with respect to the healthy one. We did not take into account further remodelling, e.g. modifications at the ionic level or the presence of BZ [25]. However, we have developed a version of our model that includes a BZ and verified in S1 Text that the GSA results do not depend on the presence of a BZ.

## Conclusions

We have shown that our model can capture the interaction between host myocardium and EHT patches by validating it against independent experimental studies. We have found that engrafting an EHT patch on non-transmural scar has no effect on electrical propagation and that, when the scar is transmural and non-conductive, the EHT patch conductivity is the main parameter influencing electrical propagation. Moreover, we demonstrated that EHT patches effects on host myocardium and EHT patch design variables are not species-dependent, providing further evidence that results in small animal model can be transferred to human models. Finally, our model indicated that, while it is possible to achieve physiological activation by tuning EHT conductivity, this approach may promote arrhythmic behavior. Instead, altering other immature characteristic of hiPSC-CMs, such as low AP upstroke velocity and lack of I_K1_, may recover physiological activation while not increasing arrhythmia initiations.

## Supporting information

S1 Text Supporting material. **Fig A:** From 3D geometry to our idealized thin 3D model. A) Human left ventricle anatomy model with transmural scar. B) Plane cutting transmurally through the ventricle wall. C) Extracted transmural section. D) Idealization of the geometry and application of EHT patch mimicking experimental design. **Fig B:** Visualization from 3 different angles of our model’s fibres distribution. A: frontal view. B: angled view. C: Side view. Our slab model is made of a layer of tetrahedral elements. A vector determining fibre orientation is assigned to each element. Fibres are rotating from endocardium to epicardium. The EHT patch is assumed isotropic. **Fig C:** Schematic representations of the models used in the validation step. The crosses indicate the nodes that were selected to extract the activation times and to compute the conduction velocities. **Fig D:** Upper panel: comparison of activation patterns in the model with the internal bath thickness set to 0.1 or 0 mm. Lower panel: table comparing the REATs for both internal bath thicknesses, for each of the 5 setups, in the rat model. **Fig E:** Comparison of sensitivity indices in the rat model (fixed sub-case) between the original model and models with REAT capped to 150, 175, and 200% of the predicted REAT without the scar. We observed a variation of 10, 1, and 3%, respectively. **Fig F:** Generation of the full 3D model (right) from the thin 3D model (left), for the human model (fixed sub-case). **Fig G:** Repolarization gradient for the full 3D model. The left panel shows the entire mesh. The right panel shows a transmural cross-section, for comparison with the repolarization gradient of the thin 3D model (Fig M in S1 Text). **Fig H:** Total effect indices comparison between the original model and the model paced from the endocardium (rat model, fixed sub-case). **Fig I:** Total effect indices comparison between original model and model paced at 2 Hz (the original model was paced at 1 Hz) (rat model, fixed sub-case). **Fig J:** Comparison of the original model (left-hand side) and the model with a BZ (right-hand side). Panel A shows schematic representations of the original model and the BZ model. Panel B shows the total effect indices of both models, for the fixed setup of the rat model. **Fig K:** Schematic representations of the human model (fixed sub-case) with transmural heterogeneity of adult ventricular cells included. The myocardium is divided into endocardium, mid-myocardium and epicardium, indicated in the figures by 3 different shades of blue, respectively from darker to lighter. **Fig L:** Total effect indices comparison between the original model and the model with transmural heterogeneities included (human model, fixed sub-case). **Fig M:** Spatial plots of the repolarization times for the human model (fixed sub-case). **Fig N:** The upper panel shows the repolarization gradient over the whole mesh at the 100th beat. In the lower panel, the blue rectangles indicate the areas considered for reporting the repolarization gradient. The values shown in Fig 7 in the main manuscript are the repolarization gradients computed for the mesh nodes located in this area. **Fig O:** Comparison of AP traces from a myocardial node close to the EHT-tissue interface and the default myocardial AP (taken from a node far from the EHT-tissue interface). The black arrows indicate the increment in APD due to the electrotonic load exerted from the EHT on the tissue at the interface. A higher EHT conductivity causes a bigger increment in APD in neighboring host myocardium, resulting in turn in a higher repolarization gradient. **Fig P:** Results obtained by modifying the EHT gNa and gK1 in the tissue (left) and cell (right) models. Left: the grid shows the REAT for the tissue model paced at 1 Hz. Values inside the blue contour are ≤ the predicted healthy REAT and thus would restore pre-infarct activation. Green squares indicate no presence of ectopy, while grey squares indicate presence of ectopy. Right: the grid shows the self-activation frequency for the hiPSC-CM cell model, in a no-pacing protocol. Red squares show where the cell model self-activates with frequency < 1 Hz (i.e., intrinsic-cycle length > 1000 ms), while grey boxes show where the cell self-activates with frequency > 1 Hz, thus causing the observed presence of ectopies in the tissue model. **Fig Q:** AP traces from a mesh node in the EHT, with different values of gNa and gK1. The tissue model is paced at 1 Hz in all 3 cases. In the baseline case (grey trace) the EHT is activated every 1000 ms by the stimulus coming from the myocardium, which is paced at 1Hz. When increasing gNa to 4 times the default value (blue trace), in order to increase the EHT CV, we observe a shortening in the intrinsic hiPSC-CM cycle length. Thus, the EHT self-activates at ~800 ms, before being stimulated by the myocardium, causing an ectopic beat. Finally, when increasing gK1 to 1.4 times the default value, the intrinsic hiPSC-CM cycle length is brought back to values > 1000 ms, allowing the EHT to be stimulated from the myocardium and thus eliminating the ectopy. **Fig R:** Snapshots from the tissue model showing the ectopic beat fired from the EHT. The snapshots are from the tissue simulation where gNa in the hiPSC-CM model was multiplied by 4. The first two rows show the normal activation and repolarization caused by the stimulus from the myocardium. At t = 500 ms, the repolarization in both the myocardium and the EHT is completed (first snapshot, third row). Around t = 800 ms, the ectopic beat is fired from the EHT (snapshot in the black rectangle). The ectopic beat can also be seen in the blue AP trace in the lower part of the figure, in contrast with the grey trace, representing the baseline (default gNa and gK1), 1 Hz activation.
(DOCX)
