# Supplementary material for: Modelling the interaction between stem cells derived cardiomyocytes patches and host myocardium to aid non-arrhythmic engineered heart tissue design
Source: PLoS Comput Biol. 2022 Apr 1;18(4):e1010030. doi: 10.1371/journal.pcbi.1010030 (PMC9007348; doi:10.1371/journal.pcbi.1010030)
Supplement: S1 Text — Fig A: From 3D geometry to our idealized thin 3D model. A) Human left ventricle anatomy model with transmural scar. B) Plane cutting transmurally through the ventricle wall. C) Extracted transmural section. D) Idealization of the geometry and application of EHT patch mimicking experimental design. Fig B: Visualization from 3 different angles of our model’s fibres distribution. A: frontal view. B: angled view. C: Side view. Our slab model is made of a layer of tetrahedral elements. A vector determining fibre orientation is assigned to each element. Fibres are rotating from endocardium to epicardium. The EHT patch is assumed isotropic. Fig C: Schematic representations of the models used in the validation step. The crosses indicate the nodes that were selected to extract the activation times and to compute the conduction velocities. Fig D: Upper panel: comparison of activation patterns in the model with the internal bath thickness set to 0.1 or 0 mm. Lower panel: table comparing the REATs for both internal bath thicknesses, for each of the 5 setups, in the rat model. Fig E: Comparison of sensitivity indices in the rat model (fixed sub-case) between the original model and models with REAT capped to 150, 175, and 200% of the predicted REAT without the scar. We observed a variation of 10, 1, and 3%, respectively. Fig F: Generation of the full 3D model (right) from the thin 3D model (left), for the human model (fixed sub-case). Fig G: Repolarization gradient for the full 3D model. The left panel shows the entire mesh. The right panel shows a transmural cross-section, for comparison with the repolarization gradient of the thin 3D model (Fig M in S1 Text). Fig H: Total effect indices comparison between the original model and the model paced from the endocardium (rat model, fixed sub-case). Fig I: Total effect indices comparison between original model and model paced at 2 Hz (the original model was paced at 1 Hz) (rat model, fixed sub-case). Fig J: Comparison of the original [file pcbi.1010030.s001.docx]

**Supplement:**

**Specifications on the tissue model**

**From 3D geometry to thin 3D idealized model:**

Fig A indicates how the model relates to a 3D experiment. As an example, we selected a human left ventricle anatomy model generated from cardiac magnetic resonance imaging[1]. To display how the thin 3D model relates to the cardiac anatomy. We have introduced a transmural scar (orange) into the human 3D left ventricle anatomy (A), we cut the ventricle to obtain a transmural section of the ventricular wall (B). We then focus on the infarcted area (C), where experimentally the engineered heart tissue (EHT) patch would be engrafted and indicate where the patch would be located relative to the scar (yellow). For our study, we have created a thin 3D model to provide a generalizable parametric representation of a patch over this transmural scar section (D). From this baseline thin 3D model, we vary the geometrical dimensions and conductivities of the different model components (scar, EHT, etc..) to predict the key design variables for determining the impact of EHT on the host-myocardium electrophysiology.


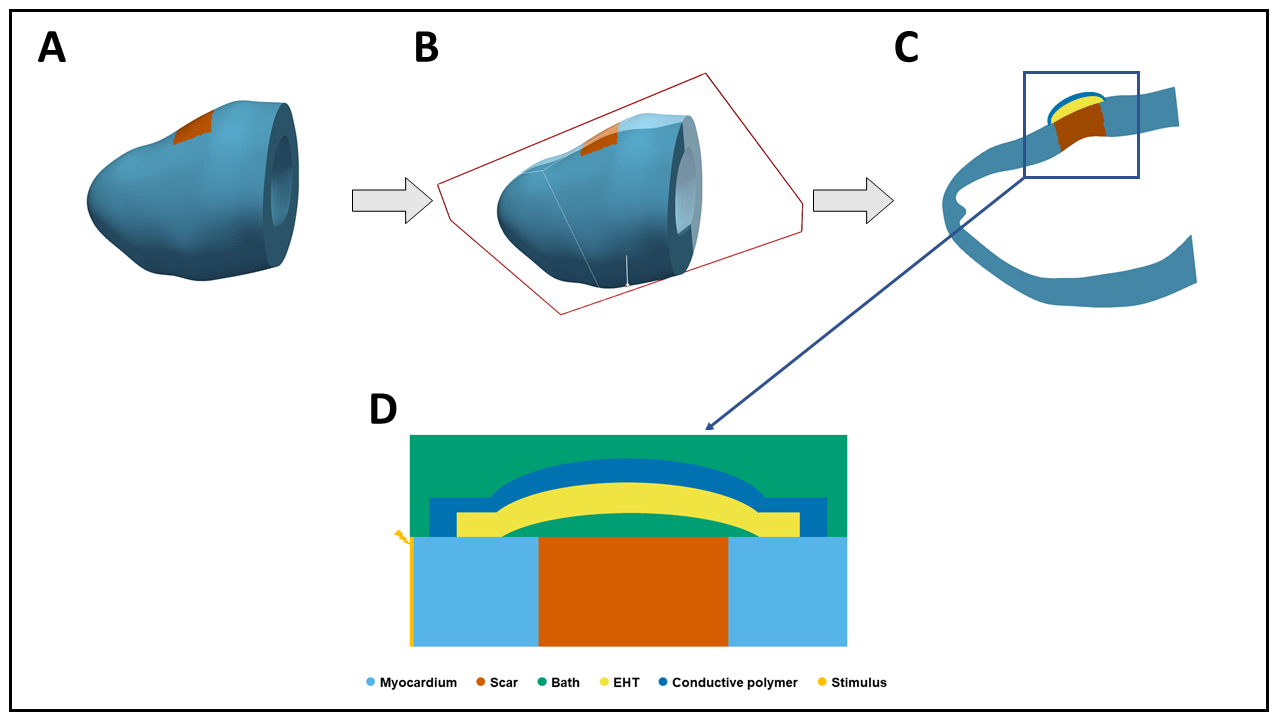


Fig A: From 3D geometry to our idealized thin 3D model. A) Human left ventricle anatomy model with transmural scar. B) Plane cutting transmurally through the ventricle wall. C) Extracted transmural section. D) Idealization of the geometry and application of EHT patch mimicking experimental design.

**Fibres orientation:**

Fig B serves as a visual aid for understanding fibres orientation in our thin 3D model. Our model is made of a layer of tetrahedral elements, and a vector determining the fibre orientation is assigned to each element. As can be seen in Fig B, the fibres transmurally rotate (from endocardium to epicardium) from a 40° angle to a -50° angle, with respect to the longitudinal direction. This approach follows a previously validated method for assigning fibres to ventricular models[2].


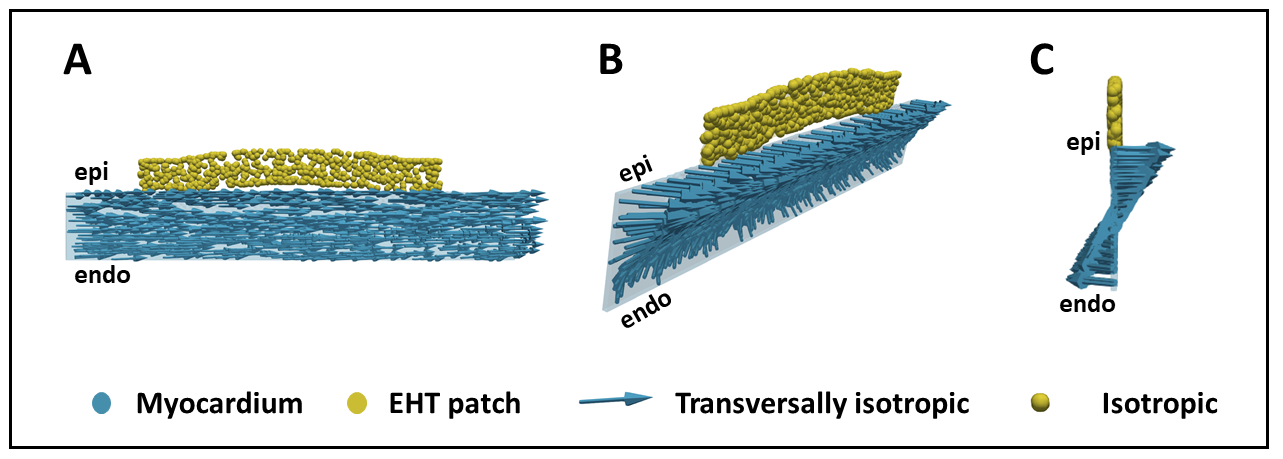


*Fig B: Visualization from 3 different angles of our model’s fibers distribution. A: frontal view. B: angled view. C: Side view. Our slab model is made of a layer of tetrahedral elements. A vector determining fibre orientation is assigned to each element. Fibers are rotating from endocardium to epicardium. The EHT patch is assumed isotropic.*

**Model validation**

**Calculation of conduction velocities:**

Fig C below shows schematic representations of the models used to run the simulations for the validation, as well as the nodes selected to compute the conduction velocities during the validation.

In Figure 2 in the manuscript, the conduction velocity (CV) between two nodes was computed by dividing their distance (difference in x coordinate, given that the nodes were selected with the same y coordinate) by the difference in the node activation times. Except when replicating the Jackman et al. experiment (Figure 2A), where the CV had to be measured at specific sites, we selected two epicardial nodes, one on the left side of the slab (before the scar) and one on the right side of the slab (after the scar), to compute a mean CV across the entire slab. The location of the selected nodes is reported below in Fig C.


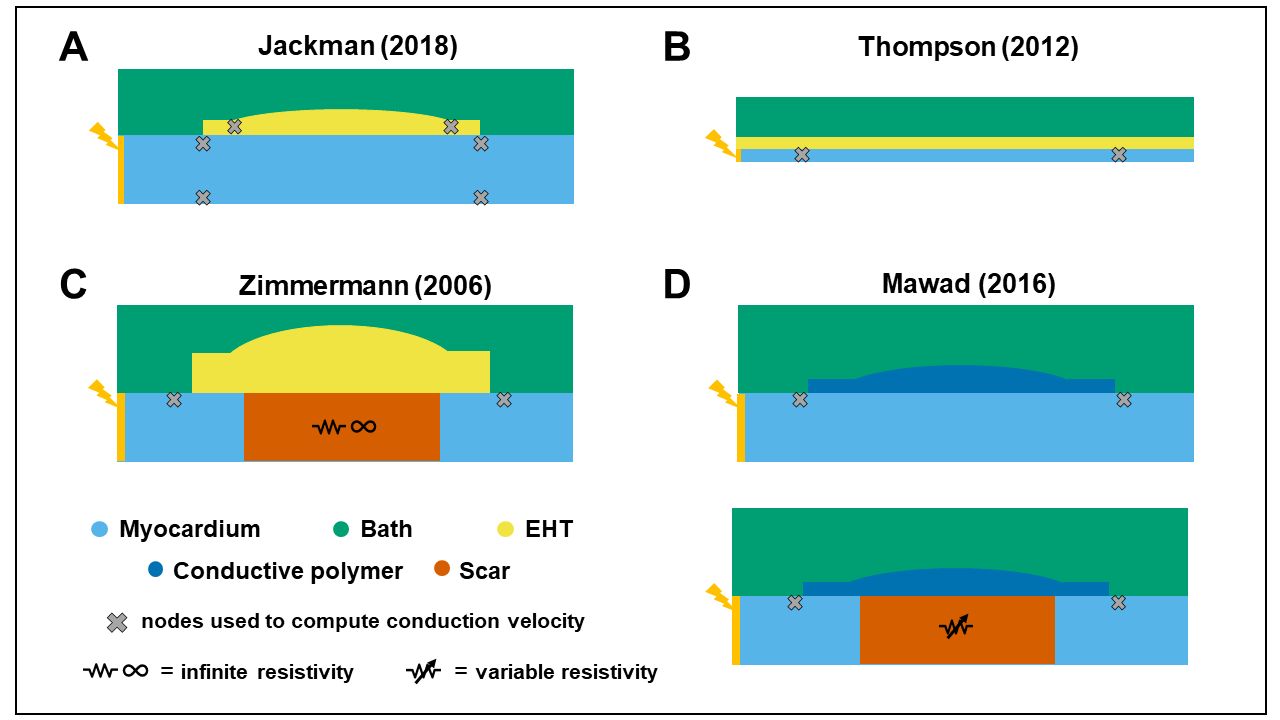


*Fig C: Schematic representations of the models used in the validation step. The crosses indicate the nodes that were selected to extract the activation times and to compute the conduction velocities.*

**Impact of internal bath absence on right epicardial activation time:**

The internal bath thickness (IBT) was set to 0 in the models used for the validation. However, in the range of parameters tested, it was always greater than 0, being the spanned interval 0.1 – 1 mm. We made this choice because: 1- we generated models automatically with dimensions described by scalars; 2- we wanted to have a consistent mesh discretization for running simulations; 3- We were concerned that meshes would fail to resolve a gap between 0 and 0.1 mm without a very high mesh resolution. For these reasons, we took a pragmatic decision and set 0.1 mm as the lower bound for the IBT with the intention of streamlining model generation and standardizing mesh generation without the need for model-specific mesh refinement.

To test if the 0.1 mm gap causes a meaningful difference in activation times relative to the 0 mm gap used in the validation simulations, we ran 5 additional simulations to compare models with IBT of 0.1 mm and 0 mm. We chose the rat model, and run simulations with a IBT of 0 mm for each sub-case (epi-endo, endo-epi, transmural, block, fixed), choosing one of the parameter combinations previously sampled, where IBT was 0.1 mm. As displayed in Fig D, the electrical propagation and the right epicardial activation time (REAT) in the compared models are almost identical, showing that including an IBT of 0 mm in the parameter range is not likely to impact the results.


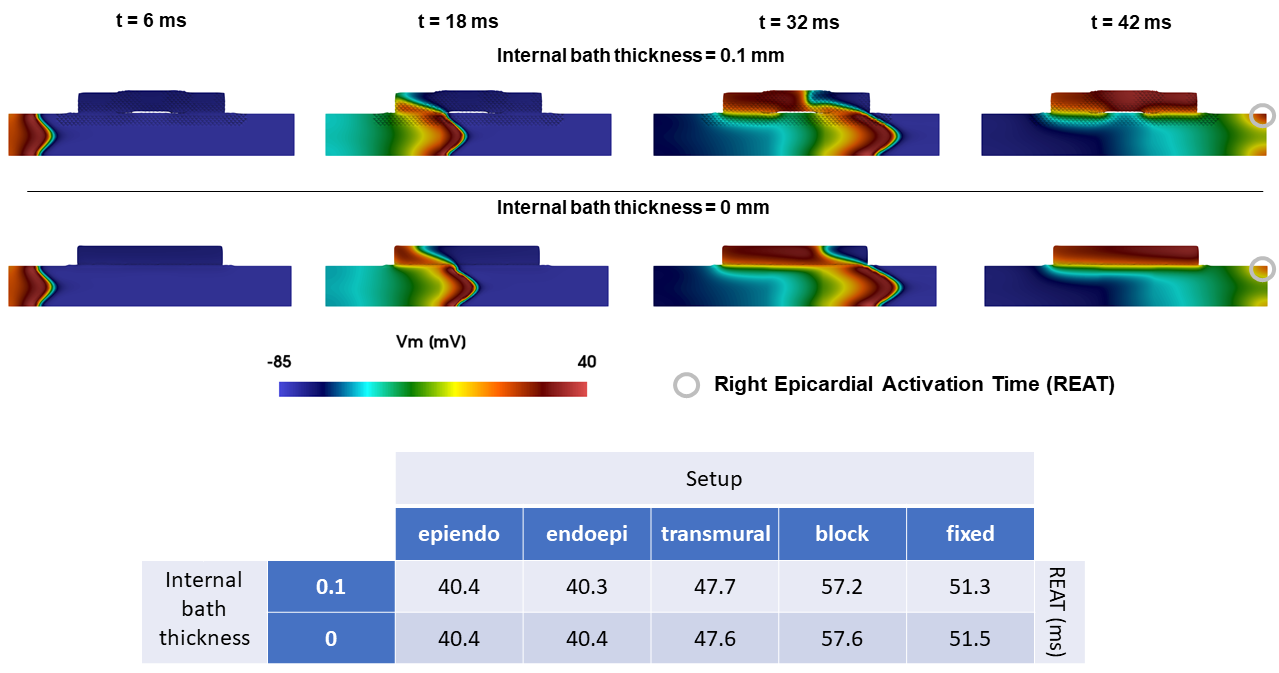


Fig D: Upper panel: comparison of activation patterns in the model with the internal bath thickness set to 0.1 or 0 mm. Lower panel: table comparing the REATs for both internal bath thicknesses, for each of the 5 setups, in the rat model.

**Impact of tridimensionality**

**Impact of tridimensionality on sensitivity analysis:**

In this study, we used an idealized thin 3D tissue model that does not include realistic whole-heart structure or geometry. Our model cannot therefore capture some phenomena arising from the 3D structure and complexity typical of the mammal ventricles (for example an electrical wave propagating around the scar rather than through the EHT patch). To test if activation waves travelling around the scar may impact our sensitivity analysis, we repeated the sensitivity analysis in the rat model (fixed sub-case), with the REAT capped to be less than 150, 175 or 200% of the predicted REAT when the scar was absent. We found a variation in the sensitivity indices of 10, 1, and 3%, when the REAT was capped, respectively, to 150, 175, and 200% of the predicted REAT without the scar (Fig E). This suggests that the results of our study still hold when considering the possibility that the activation wave propagates around the scar.


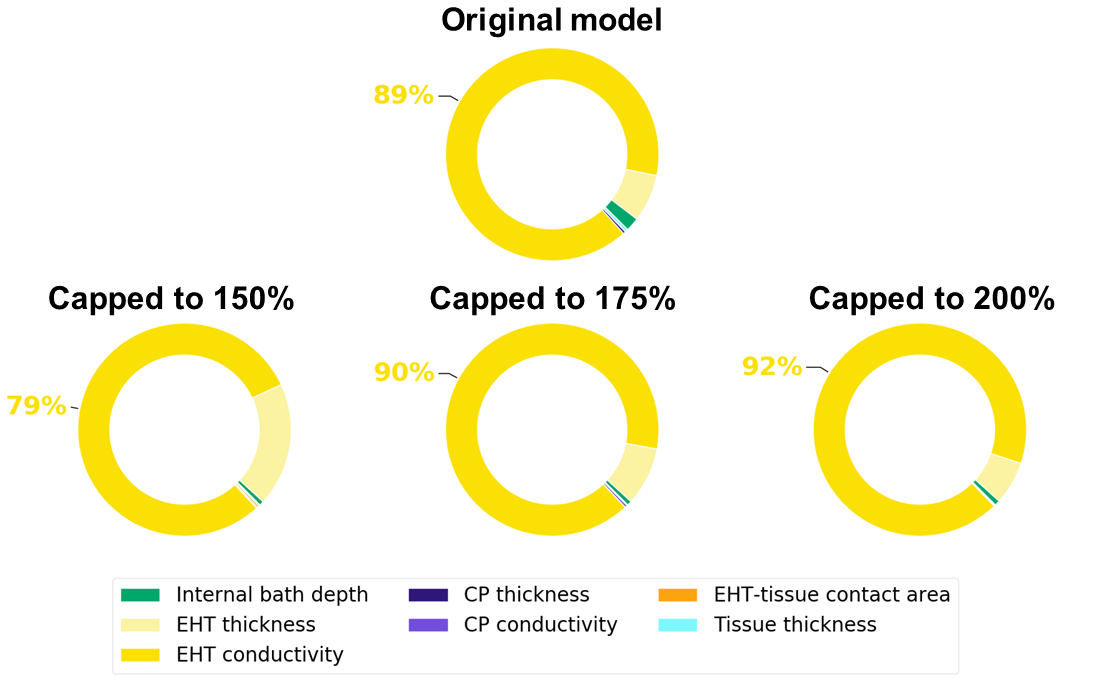


*Fig E: Comparison of sensitivity indices in the rat model (fixed sub-case) between the original model and models with REAT capped to 150, 175, and 200% of the predicted REAT without the scar. We observed a variation of 10, 1, and 3%, respectively.*

**Impact of tridimensionality on repolarization gradients:**

We used a thin 3D model in our primary analysis, which may not account for the impact on the repolarization gradients of different activation paths and scar morphologies typical of a 3D environment. To investigate whether dimensionality could affect the repolarization gradient, we computed the repolarization gradient for propagation simulated in a full 3D version of our human mode (fixed sub-case). The full 3D model was created by extending the current thin 3D model in the z-direction (Fig F), i.e., the full 3D model featured multiple layers of elements in the z-direction, as opposed to only one layer of elements featured by the original, thin 3D model.


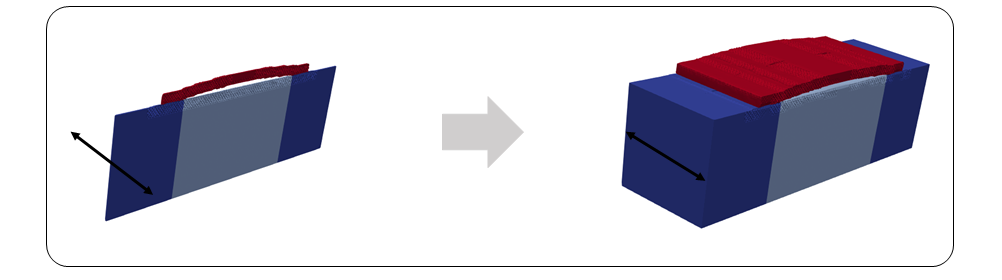


*Fig F: Generation of the full 3D model (right) from the thin 3D model (left), for the human model (fixed sub-case).*

Fig G shows the repolarization gradient in the full 3D model. Comparing it with the repolarization gradient of the thin 3D model in Figure 3 reveals that their magnitudes are similar (maximum values ~70 ms/mm in both cases) and that the spatial distributions are also comparable, with both exhibiting higher values at the tissue-EHT interface. Thus, dimensionality has a limited impact on our model.


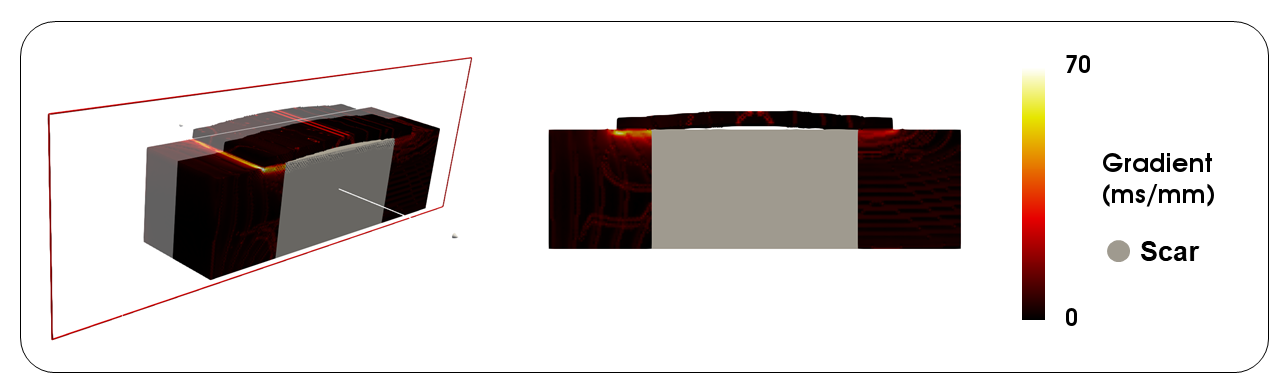


Fig G: Repolarization gradient for the full 3D model. The left panel shows the entire mesh. The right panel shows a transmural cross-section, for comparison with the repolarization gradient of the thin 3D model (Fig M).

**Testing model assumptions and parameters choice**

When creating our model and conducting this study, we made a number of assumptions and parameters choice, as it happens for every modelling study. To test if these choices impacted our results, we have repeated our Global Sensitivity Analysis (GSA) using the rat model, *fixed* sub-case as a reference model. We have tested whether our results are affected by: 1- Pacing location; 2- Pacing frequency; 3- Presence of border zone (BZ); 4- Transmural heterogeneity of ventricle adult cardiomyocytes.

1. **Pacing location:**

The model aimed to represent the common experimental set up where the heart is often paced from an electrode placed on the ventricular epicardium. To test if the pacing protocol caused a large impact on the sensitivity analysis, we repeated the sensitivity analysis on the rat *fixed* sub-case, stimulated from a point on the endocardium. This caused a change in sensitivity of 2% (Fig H).


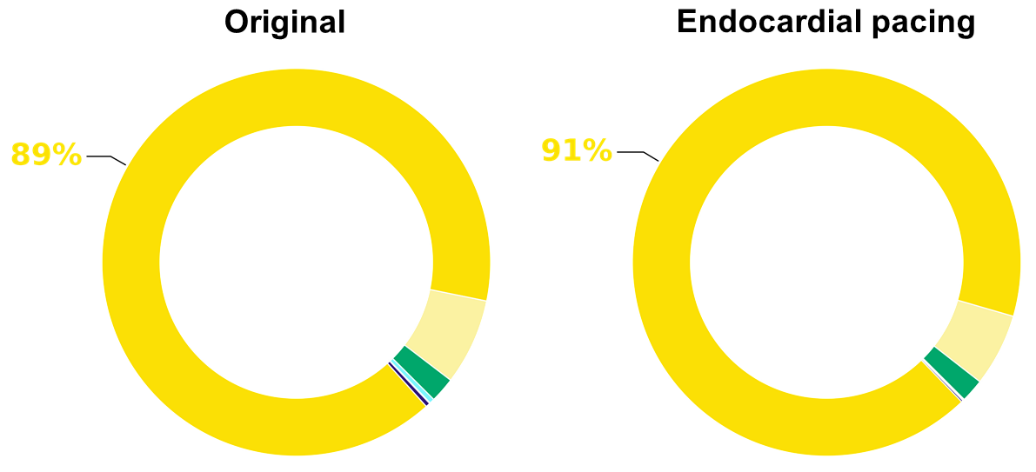


Fig H: Total effect indices comparison between the original model and the model paced from the endocardium (rat model, fixed sub-case).

1. **Pacing frequency:**

To test the output of our model at higher frequencies, we paced the TNCHS and Paci cell models with a 30 µA/cm2 transmembrane current for 800 s at 2 Hz, and we used the state variables to initialize the cells model in the tissue simulation. We repeated the 500 simulations (with the same parameters combinations sampled previously) for the rat fixed model, and performed the sensitivity analysis on the new simulation results. The comparison shows a 1% variation in the sensitivity indices, thus demonstrating that our results are not affected when pacing the model faster than 2 Hz.


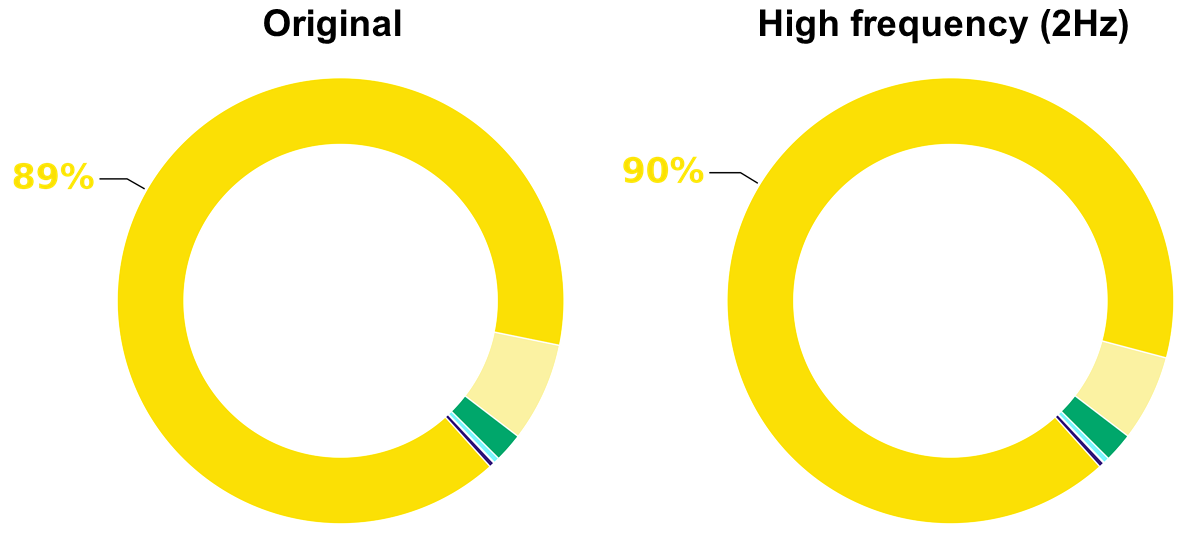


Fig I: Total effect indices comparison between original model and model paced at 2 Hz (the original model was paced at 1 Hz) (rat model, fixed sub-case).

1. **Presence of Border Zone:**

In this section we create and test a version of our slab model featuring a border zone (BZ) around the scar. The BZ replaces a portion of the scar in the original model. Specifically, the BZ thickness corresponds to 10% of the original scar width (see schematic representation in Fig J-A). For example, if the original scar width was 6 mm, a border of 0.6 mm thickness will be assigned as the BZ. Isotropic tissue was assigned in the BZ, to account for structural remodeling and fibers disarray, with a conductivity set to 50% of the healthy transversal conductivities[3].

We repeated the 500 simulations (with the same parameters combinations sampled previously) for the fixed setups of the rat model. We also trained Gaussian processes emulators (GPEs) for both setups and performed the GSA. A comparison of the total effect indexes between the models without and with BZ is reported in Fig J-B, showing a 1% change.

*
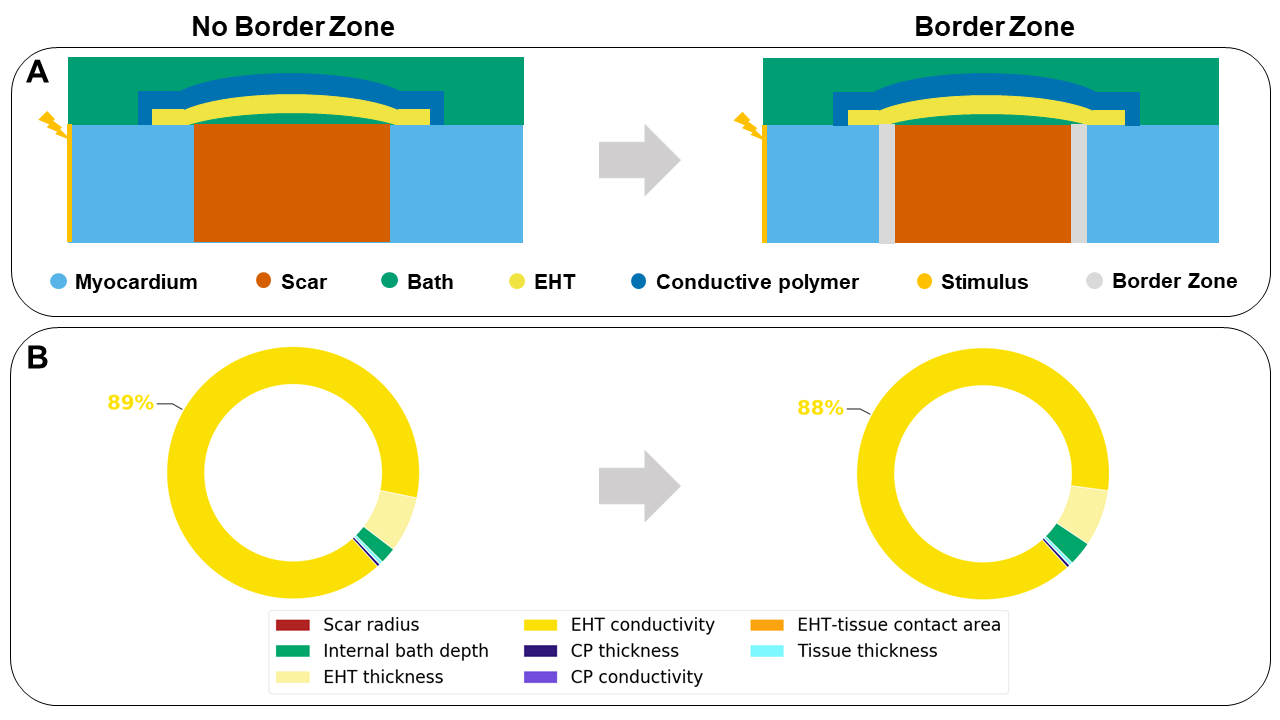

Fig J: Comparison of the original model (left-hand side) and the model with a BZ (right-hand side). Panel A shows schematic representations of the original model and the BZ model. Panel B shows the total effect indices of both models, for the fixed setup of the rat model.*

1. **Transmural heterogeneity of adult ventricular myocytes:**

Transmural heterogeneities of adult cells were not included in our tissue model. The rat cell model and the rabbit model that we used did not have transmural versions, and creating calibrated endo and epicardial versions of these models was outside the scope of this study. However, to estimate the impact of transmural heterogeneity on tissue activation, we developed a version of the model where we defined three different transmural layers for endocardial, mid-myocardial and epicardial myocytes spanning 17, 41, and 42% of our model wall thickness, respectively (see Fig K).


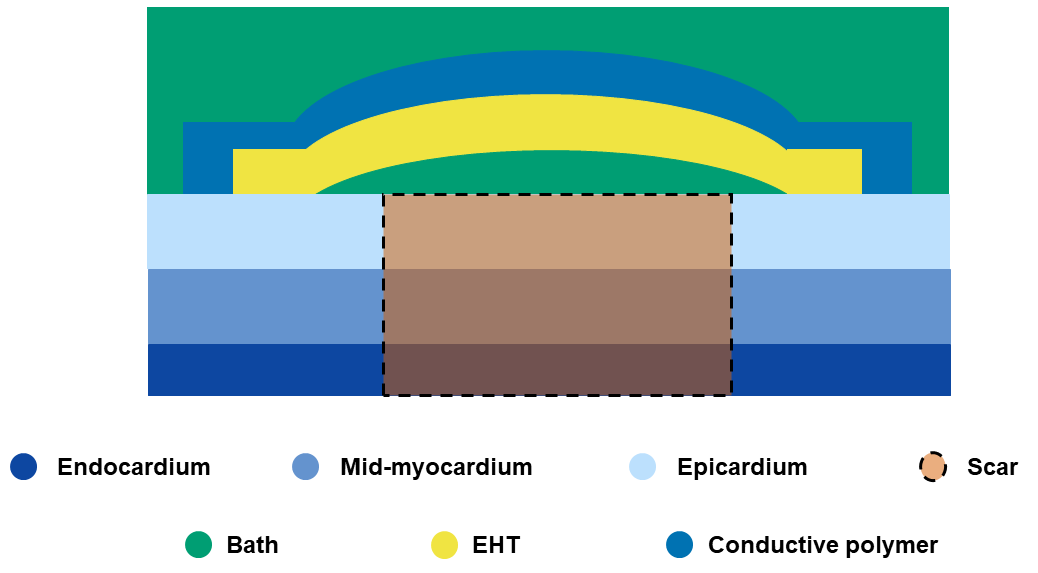


Fig K: Schematic representations of the human model (fixed sub-case) with transmural heterogeneity of adult ventricular cells included. The myocardium is divided into endocardium, mid-myocardium and epicardium, indicated in the figures by 3 different shades of blue, respectively from darker to lighter.

These values were estimated from the data reported by several experimental studies[4–6]. The layers where then assigned with the endocardial, mid-myocardial and epicardial version of the Ten Tusscher ionic model. Comparison of sensitivity indices for the human model (*fixed* sub-case) shows that including the transmural heterogeneity of adult human cells does not influence our results, causing a maximum change in sensitivity of 3% (Fig L).


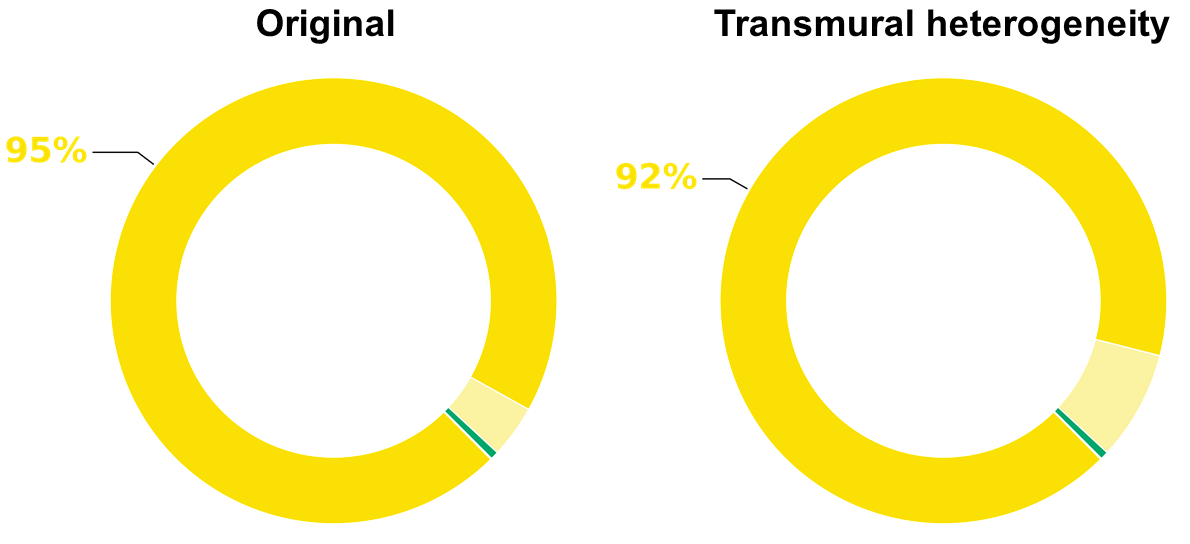


*Fig L: Total effect indices comparison between the original model and the model with transmural heterogeneities included (human model, fixed sub-case).*

**Specifications on Repolarization gradients**

**Repolarization gradients calculation:**

To compute the repolarization gradients, we paced the model for 100 beats to reduce any transient effects. For the 100th beat only, we calculated the time to 80% repolarization at each node of the mesh. An example of repolarization times spatial plot is reported in Fig M.


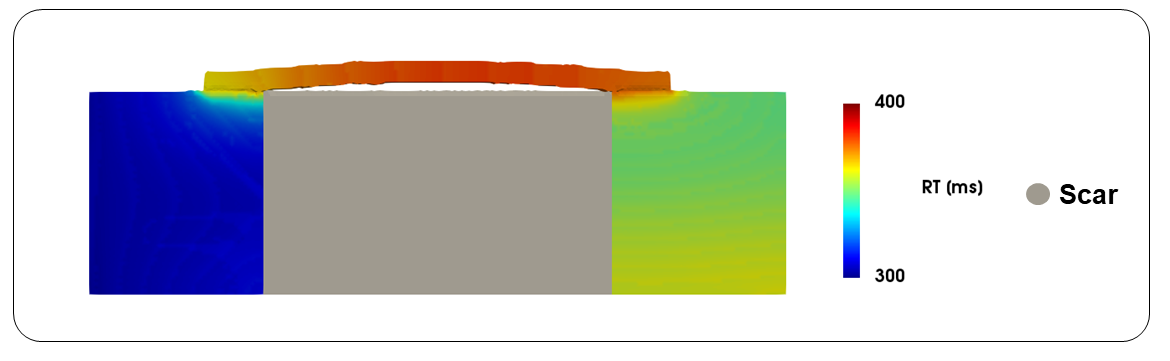


Fig M: Spatial plots of the repolarization times for the human model (fixed sub-case).

We then calculated the magnitude of the gradient of these repolarization times. Fig N displays the repolarization gradients on the mesh and shows which nodes were considered when reporting the repolarization gradient in Figure 7 in the main manuscript. We considered the nodes located in the area depicted in the figure with a light green rectangle. The edge length of the rectangle base equals the EHT-myocardium contact area, while the rectangle height equals 1 mm, to focus on the myocardial area closer to the EHT.

We focused on this area as it was the area with the highest gradients, it was the area most affected by the presence of the EHT and was close to the border of the scar, which is an area that is prone to ectopics[7]. Repolarization gradients can be used as a surrogate for arrhythmia marker because a high repolarisation gradient implies there is a greater chance of an ectopic beat generating arrhythmogenic unidirectional block[8].


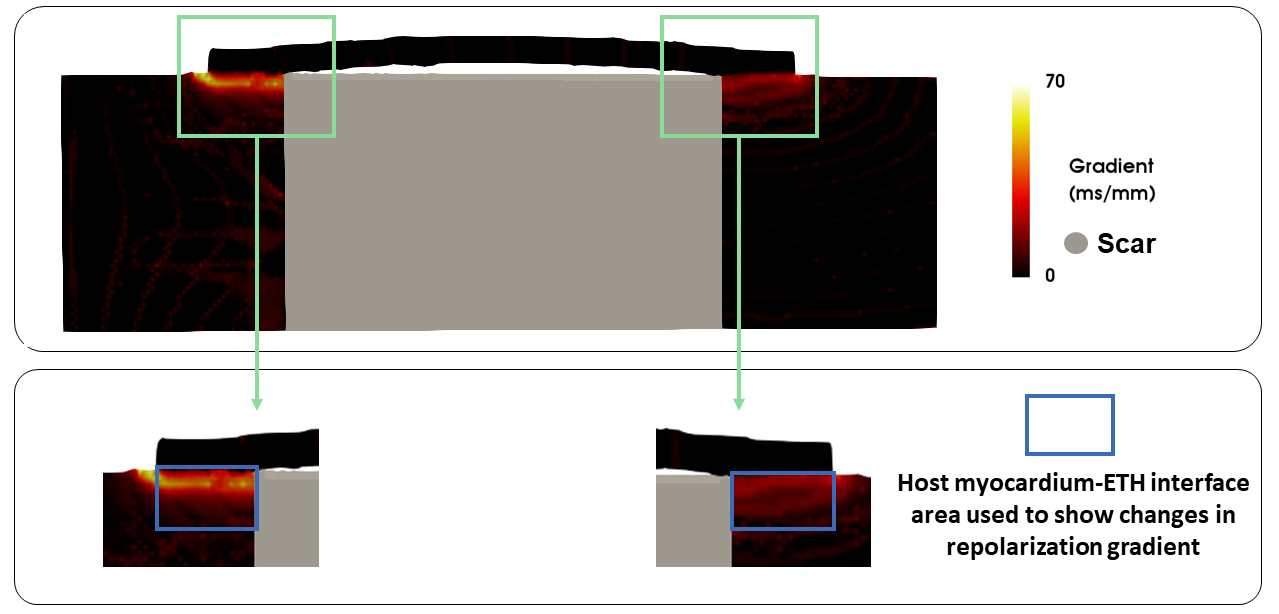


*Fig N: The upper panel shows the repolarization gradient over the whole mesh at the 100th beat. In the lower panel, the blue rectangles indicate the areas considered for reporting the repolarization gradient. The values shown in Figure 7 in the main manuscript are the repolarization gradients computed for the mesh nodes located in this area.*

**Effect of EHT conductivity on repolarization gradients:**

Increasing EHT tissue conductivity increases repolarization gradients at the interface because it increases the electrotonic load the EHT exerts on the host myocardium. The electrotonic load brings the APD of the host myocardium (~310 ms) closer to the EHT APD (~ 420 ms). A higher EHT conductivity causes a higher electrotonic load, leading in turn to a more prominent lengthening of the host myocardium APD (Fig O). Finally, a greater APD lengthening leads to higher repolarization gradients. The following figure (Fig O) shows how the AP of a myocardial node at the tissue-EHT interface changes when changing the conductivity in the EHT.


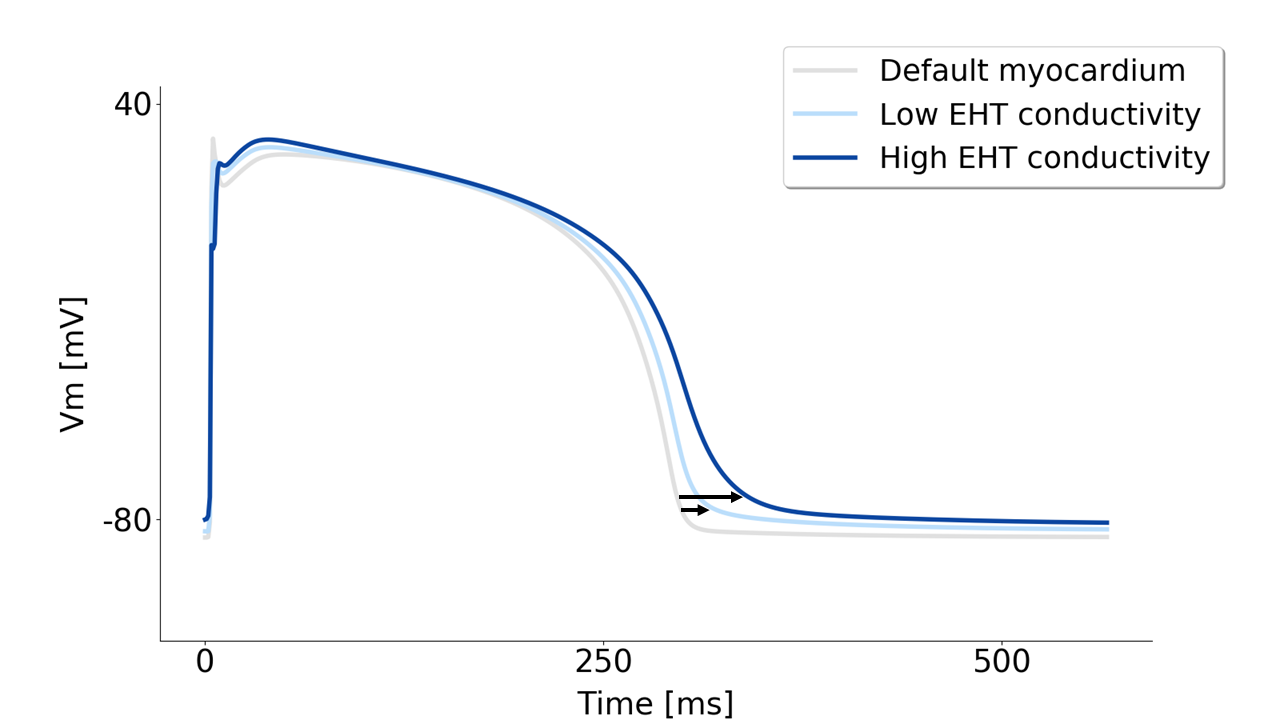


Fig O: Comparison of AP traces from a myocardial node close to the EHT-tissue interface and the default myocardial AP (taken from a node far from the EHT-tissue interface). The black arrows indicate the increment in APD due to the electrotonic load exerted from the EHT on the tissue at the interface. A higher EHT conductivity causes a bigger increment in APD in neighboring host myocardium, resulting in turn in a higher repolarization gradient.

**How changing Paci model parameters (gNa and gK1) affects the EHT action potential and the presence of ectopies in the EHT**

To increase the CV in the EHT without increasing the conductivity, we increased the Paci model parameter (gNa), which models the density of ion channels related to the fast sodium current. Although we succeeded in recovering a healthy REAT, increasing gNa resulted in a shortening of the intrinsic hiPSC-CM cycle-length down to 806 ms (1.24 Hz), in turn causing the firing of ectopic beats from the EHT when the tissue was paced at 1 Hz.

Ectopies essentially arise because, in the hiPSC-CM with increased gNa, the transmembrane potential increases more rapidly after repolarization, causing a more frequent repetition of the intrinsic activation cycle. To slow the intrinsic activation of the hiPSC-CM and to allow the EHT to be activated by the myocardium, we increased the Paci model parameter (gK1) modeling the density of ion channels related to the inward rectifier potassium current.

After running our tissue model with 3, 3.25. 3.5, 3.75, 4 and 4.25 as multipliers for the default Paci model gNa, and 1.1, 1.2, 1.3, 1.4 and 1.5 as multipliers for the default Paci model gK1, we show (Fig P – left panel) that multipliers equal or greater than 4 and 1.4 (for gNa and for gK1, respectively) are needed to recover a healthy REAT while avoiding EHT-generated ectopies at 1 Hz pacing.

We also ran single-cell simulations using the hiPSC-CM ionic model (Paci model) and looked at the intrinsic single-cell activation rate for each parameter combination used in the tissue model. We found that the intrinsic activation frequency dropped below 1 Hz for the same parameter combinations for which no ectopies were observed in the tissue model simulations (paced at 1Hz).


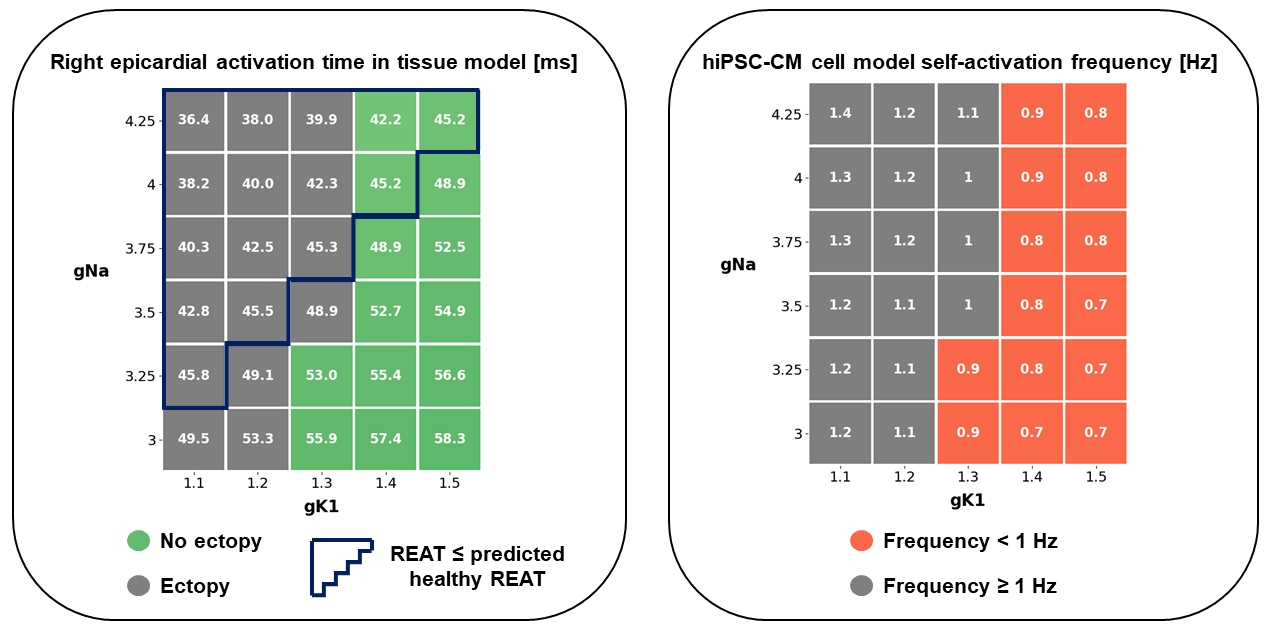


Fig P: Results obtained by modifying the EHT gNa and gK1 in the tissue (left) and cell (right) models. Left: the grid shows the REAT for the tissue model paced at 1 Hz. Values inside the blue contour are ≤ the predicted healthy REAT and thus would restore pre-infarct activation. Green squares indicate no presence of ectopy, while grey squares indicate presence of ectopy. Right: the grid shows the self-activation frequency for the hiPSC-CM cell model, in a no-pacing protocol. Red squares show where the cell model self-activates with frequency < 1 Hz (i.e., intrinsic-cycle length > 1000 ms), while grey boxes show where the cell self-activates with frequency > 1 Hz, thus causing the observed presence of ectopies in the tissue model.

The AP traces reported below in Fig Q serve as a visual aid for understanding how the EHT electrophysiology is affected when changing gNa and gK1. Increasing gNa produces an increase in the EHT AP upstroke, and thus in the CV in the EHT. However, increasing gNa also shortens the intrinsic hiPSC-CM cycle length, resulting in ectopic beats fired from the EHT when pacing tissue at 1 Hz (blue trace in Fig Q). Increasing gK1 slows down the intrinsic activation of the hiPSC-CM and allows the EHT to be activated by the myocardium, paced at 1 Hz (orange trace in Fig Q).


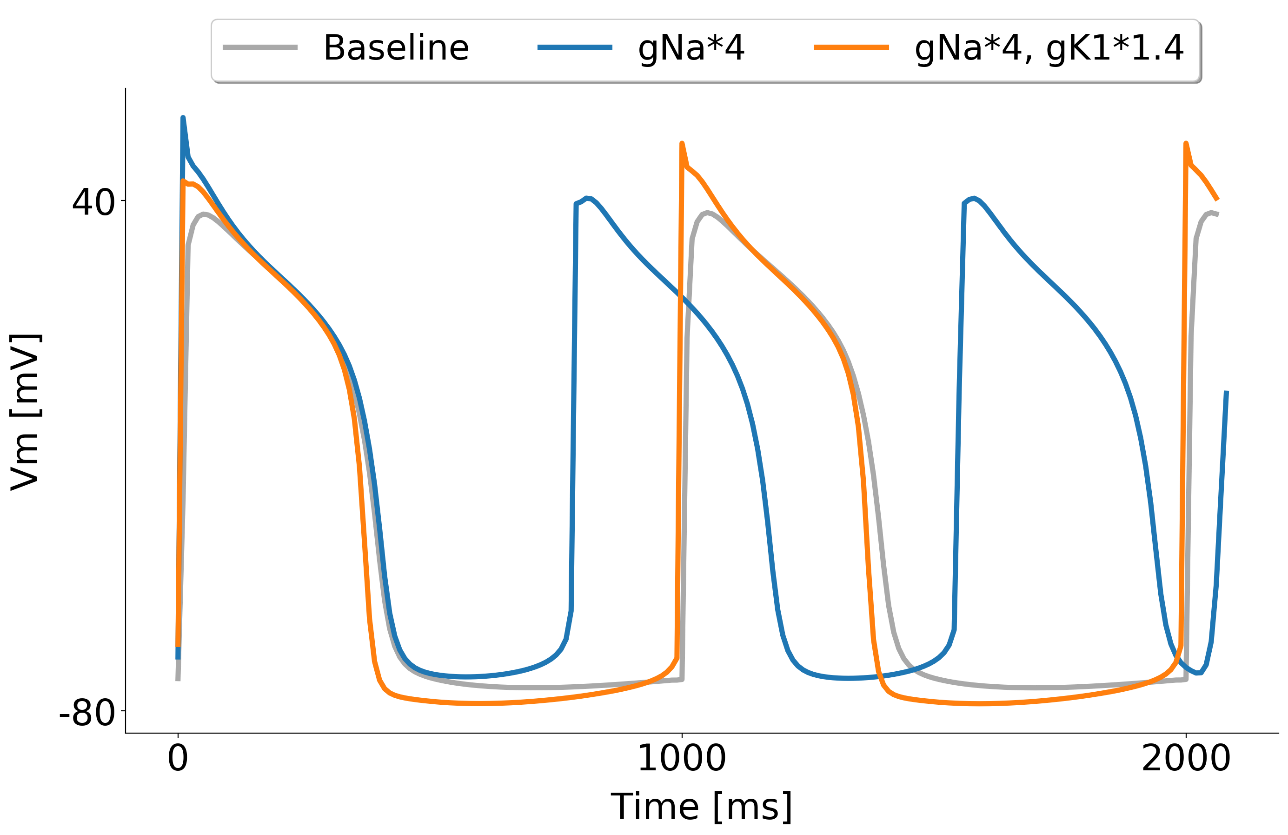


*Fig Q: AP traces from a mesh node in the EHT, with different values of gNa and gK1. The tissue model is paced at 1 Hz in all 3 cases. In the baseline case (grey trace) the EHT is activated every 1000 ms by the stimulus coming from the myocardium, which is paced at 1Hz. When increasing gNa to 4 times the default value (blue trace), in order to increase the EHT CV, we observe a shortening in the intrinsic hiPSC-CM cycle length. Thus, the EHT self-activates at ~800 ms, before being stimulated by the myocardium, causing an ectopic beat. Finally, when increasing gK1 to 1.4 times the default value, the intrinsic hiPSC-CM cycle length is brought back to values > 1000 ms, allowing the EHT to be stimulated from the myocardium and thus eliminating the ectopy*

We also provide in Fig R below snapshots of the tissue model showing the ectopic beats firing from the EHT, and the corresponding AP traces for a mesh node in the EHT.


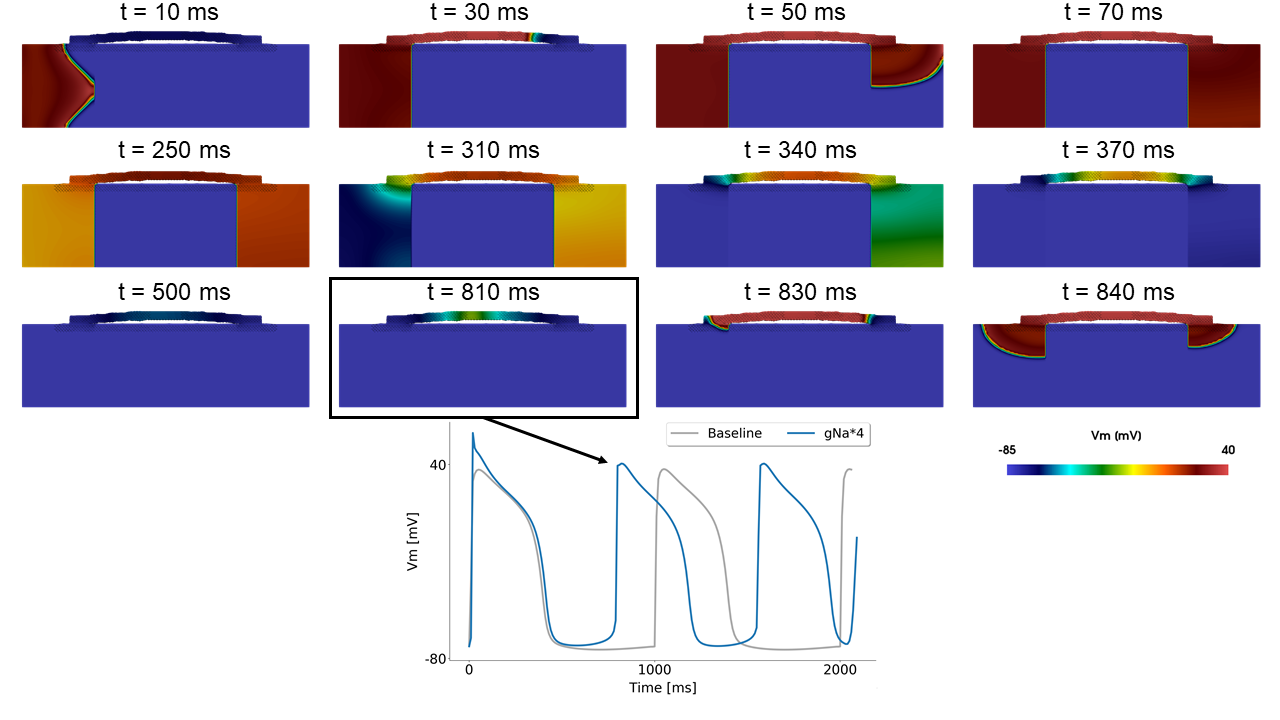


*Fig R: Snapshots from the tissue model showing the ectopic beat fired from the EHT. The snapshots are from the tissue simulation where gNa in the hiPSC-CM model was multiplied by 4. The first two rows show the normal activation and repolarization caused by the stimulus from the myocardium. At t=500 ms, the repolarization in both the myocardium and the EHT is completed (first snapshot, third row). Around t = 800 ms, the ectopic beat is fired from the EHT (snapshot in the black rectangle). The ectopic beat can also be seen in the blue AP trace in the lower part of the figure, in contrast with the grey trace, representing the baseline (default gNa and gK1), 1 Hz activation.*

**References**

1. Mendonca Costa C, Neic A, Kerfoot E, Gillette K, Porter B, Sieniewicz B, Gould J, Sidhu B, Chen Z, Elliott M, Mehta V, Plank G, Rinaldi A, Bishop M NS. A Virtual Cohort of Twenty-four Left-ventricular Models of Ischemic Cardiomyopathy Patients. doi:https://doi.org/10.18742/RDM01-570

2. Bayer JD, Blake RC, Plank G, Trayanova NA. A novel rule-based algorithm for assigning myocardial fiber orientation to computational heart models. Ann Biomed Eng. 2012;40: 2243–2254. doi:10.1007/s10439-012-0593-5

3. Costa CM, Plank G, Rinaldi CA, Niederer SA, Bishop MJ. Modeling the electrophysiological properties of the infarct border zone. Front Physiol. 2018;9: 1–14. doi:10.3389/fphys.2018.00356

4. Yan GX, Shimizu W, Antzelevitch C. Characteristics and distribution of M cells in arterially perfused canine left ventricular wedge preparations. Circulation. 1998;98: 1921–1927. doi:10.1161/01.CIR.98.18.1921

5. Antzelevitch C, Sicouri S, Litovsky SH, Lukas A, Krishnan SC, Diego JM Di, et al. Heterogeneity Within the Ventricular Wall. Circ Res. 1991;69: 1427–1449.

6. Sicouri S, Fish J, AntzelevItch C. Distribution of M Cells in the Canine Ventricle. J Cardiovasc Electrophysiol. 1994;5: 824–837. doi:10.1111/j.1540-8167.1994.tb01121.x

7. Marrouche NF, Verma A, Wazni O, Schweikert R, Martin DO, Saliba W, et al. Mode of initiation and ablation of ventricular fibrillation storms in patients with ischemic cardiomyopathy. J Am Coll Cardiol. 2004;43: 1715–1720. doi:10.1016/j.jacc.2004.03.004

8. Laurita KR, Rosenbaum DS. Interdependence of modulated dispersion and tissue structure in the mechanism of unidirectional block. Circ Res. 2000;87: 922–928. doi:10.1161/01.RES.87.10.922
